# Trends and future projections of cancer prevalence in patients with cardiovascular admissions

**DOI:** 10.1093/ehjopen/oeag030

**Published:** 2026-02-19

**Authors:** Guia Ferrari Ardicini, Andrija Matetic, Evangelos Kontopantelis, Alaide Chieffo, Carmen Maria Moldovan, Zahra Raisi-Estabragh, Elad Asher, Christian Mallen, Mamas A Mamas

**Affiliations:** Keele Cardiovascular Research Group, Centre for Prognosis Research, Keele University, Keele ST5 5BG, UK; Vita-Salute San Raffaele University, Via Olgettina 58, 20132 Milan, Italy; Keele Cardiovascular Research Group, Centre for Prognosis Research, Keele University, Keele ST5 5BG, UK; Department of Cardiology, University Hospital of Split, Spinciceva 1, 21000 Split, Croatia; Division of Informatics, Imaging and Data Science, The University of Manchester, Oxford Road, Manchester M13 9PL, UK; Division of Family Medicine, Yong Loo Lin School of Medicine, National University of Singapore, 10 Medical Drive, 119228 Singapore, Singapore; Vita-Salute San Raffaele University, Via Olgettina 58, 20132 Milan, Italy; Interventional Cardiology Unit, IRCCS San Raffaele Scientific Institute, Via Olgettina 60, 20132 Milan, Italy; The 3rd Department of Cardiology, University of Athens, Sotiria Hospital, Mesogeion Avenue 152, 11527 Athens, Greece; Centre for Advanced Cardiovascular Imaging, William Harvey Research Institute, Queen Mary University of London, NIHR Barts Biomedical Research Centre, Barts Heart Centre, Barts Health NHS Trust, Charterhouse Square, London EC1M 6BQ, UK; Jesselson Heart Center, Shaare Zedek Medical Center, The Eisenberg R&D Authority, and Faculty of Medicine, Hebrew University of Jerusalem, 12 Bayit Street, Jerusalem 91031, Israel; School of Medicine, Keele University, Keele, Staffordshire ST5 5BG, UK; Keele Cardiovascular Research Group, Centre for Prognosis Research, Keele University, Keele ST5 5BG, UK; Royal Stoke University Hospital, Newcastle Road, Stoke-on-Trent ST4 6QG, UK; National Institute for Health and Care Research (NIHR), Birmingham Biomedical Research Centre, Mindelsohn Way, Birmingham B15 2TQ, UK

**Keywords:** Cancer, Cardiovascular admissions, Trends

## Abstract

**Aims:**

Patients with cancer have an increased risk of cardiovascular (CV) events, although there is limited data on future trends in cancer prevalence amongst patients with an acute cardiovascular admission. The aim of this study was to evaluate trends in cancer prevalence among CV admissions with an attempt to predict future cancer and CV co-morbidity over the next 20 years.

**Methods and results:**

The analysis included all hospital admissions with a primary CV diagnosis from the US National Inpatient Sample (NIS), from 2016 to 2020. The sample was stratified by specific CV admission and by cancer status and type. The chi-square and the Kruskal–Wallis tests were used to compare categorical and continuous data, respectively, across the years. A Poisson regression model was used to predict the prevalence of overall and specific cancer types through 2040, based on the 5-year baseline period. Among 4.79 million CV admissions from 2016 to 2020, there was a significant increase in cancer prevalence from 4.8% to 5.4% (*P* < 0.001). This upward trend was observed across all CV diagnoses. Predictive modelling estimates that cancer prevalence in CV inpatients will increase from a 4.8% baseline in 2016 to 11.9% by 2040, with the most pronounced rate of growth seen in liver (IRR 1.069; *P* < 0.001), breast (IRR 1.056; *P* < 0.001), and renal cancer (IRR 1.055; *P* < 0.001). Nevertheless, haematological and lung cancers show the highest prevalence, both at baseline and in 2040.

**Conclusion:**

The prevalence of cancer among patients hospitalized with CV disease is predicted to increase 2.48-fold by 2040. This trend highlights the importance of integrated cardio-oncology and multidisciplinary care models.

## Graphical Abstract

**Graphical Abstract:**
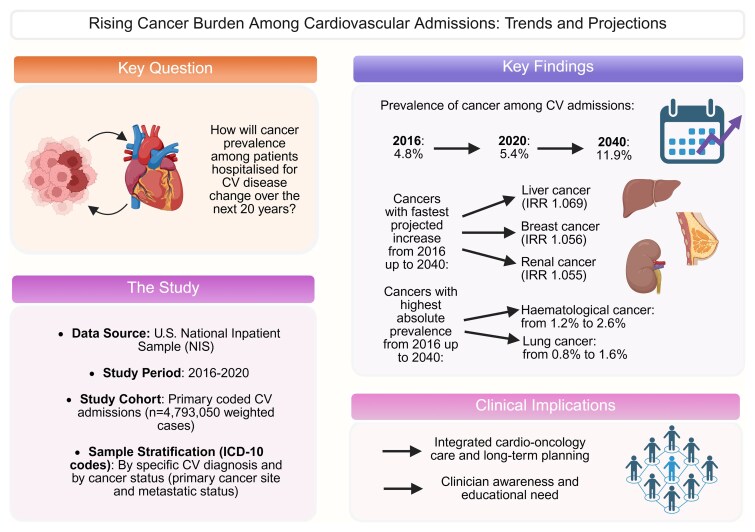

## Introduction

The overlap between cardiovascular (CV) disease and cancer poses a significant challenge to healthcare systems globally. CV disease and cancer are leading drivers of mortality worldwide,^1^ with a strong bidirectional relationship. Both CV disease and cancer potentially increase the risk of each other through shared risk factors, chronic disease burden, and common pathophysiological pathways.^2^ With innovative therapeutic options available, mortality in patients diagnosed with cancer has declined, yielding an increased number of cancer survivors,^3^ who are at increased risk of CV morbidity and mortality. Among cancer survivors, CV disease is the major cause of non-cancer death^4^ with CV mortality overtaking cancer mortality in the longer term.^5^

How this growing burden of CV disease in patients with cancer will evolve over the next few decades is unclear. Whilst the prevalence of cancer in patients with a primary CV diagnosis has been estimated to be in the order of ∼4.5%, with haematological malignancy and lung cancer being the most prevalent,^6,7^ there is a significant literature gap on their future projections. Understanding cancer prevalence and its trends among patients with CVD is therefore essential for proper healthcare planning, education and policy development.

Therefore, this study aimed to determine the baseline prevalence of cancer and its specific types in patients with acute cardiovascular admissions. Importantly, this analysis aimed to determine the future predictions and trends in cancer prevalences within this patient population over the next 20 years. Finally, we aimed to determine the association of cancer prevalence, patient characteristics and future trends across different specific cardiovascular admission causes.

## Methods

### Ethical and reporting considerations

This study was conducted using publicly available data from the National Inpatient Sample (NIS) database, which is de-identified and anonymized, ensuring compliance with the Health Insurance Portability and Accountability Act (HIPAA). This provides exemption from approval by institutional boards or ethics committees. Moreover, the manuscript adheres to the *Strengthening The Reporting of Observational Studies in Epidemiology (STROBE*) guidelines for reporting observational studies (see Supplementary material online, *Table S1*).

### Setting, participants and data sources

The NIS database was developed by the Agency for Healthcare Research and Quality (AHRQ) under the Healthcare Cost and Utilization Project (HCUP).^8^ It generates nationally representative estimates of inpatient utilization, access, charges, quality, and outcomes in the United States (US).^8^ The NIS contains anonymized discharge data from more than seven million hospitalizations yearly, representing the largest publicly available all-payer inpatient database in the US.

The analysis included all adult cases (≥18 years) with any primary cardiovascular (CV) admission, between January 2016 and December 2020, defined using the International Classification of Diseases—10th Edition (ICD-10) [Supplementary material online, *Table S1*]. The CV admissions were further categorized to focus on the most clinically relevant diagnoses that can be reliably ascertained from clinical coding systems into acute coronary syndrome (ACS), heart failure (HF), atrial fibrillation/flutter, other arrhythmias, hypertension and its complications (excluding heart failure), pulmonary embolism (PE), ischaemic stroke, haemorrhagic stroke, valve disorders, and other CV causes. Furthermore, the study sample was stratified by cancer status and further categorized by primary cancer site (based on ICD-10 codes): Breast cancer, colorectal cancer, renal/urinary cancer, liver cancer, haematological cancer, lung/bronchus cancer, prostate and male genital cancer, pancreatic cancer, female genital cancer, skin cancer, and gastroesophageal cancer. Cancer cases were also stratified by metastatic status. It should be noted that the NIS does not contain clinical information to differentiate active cancer from cancer in remission. In this study, cancer status was defined using ICD-10 C-codes, which reflect current or recent malignancy. Z-codes (personal history of malignancy) were not included.

**Figure 1:**
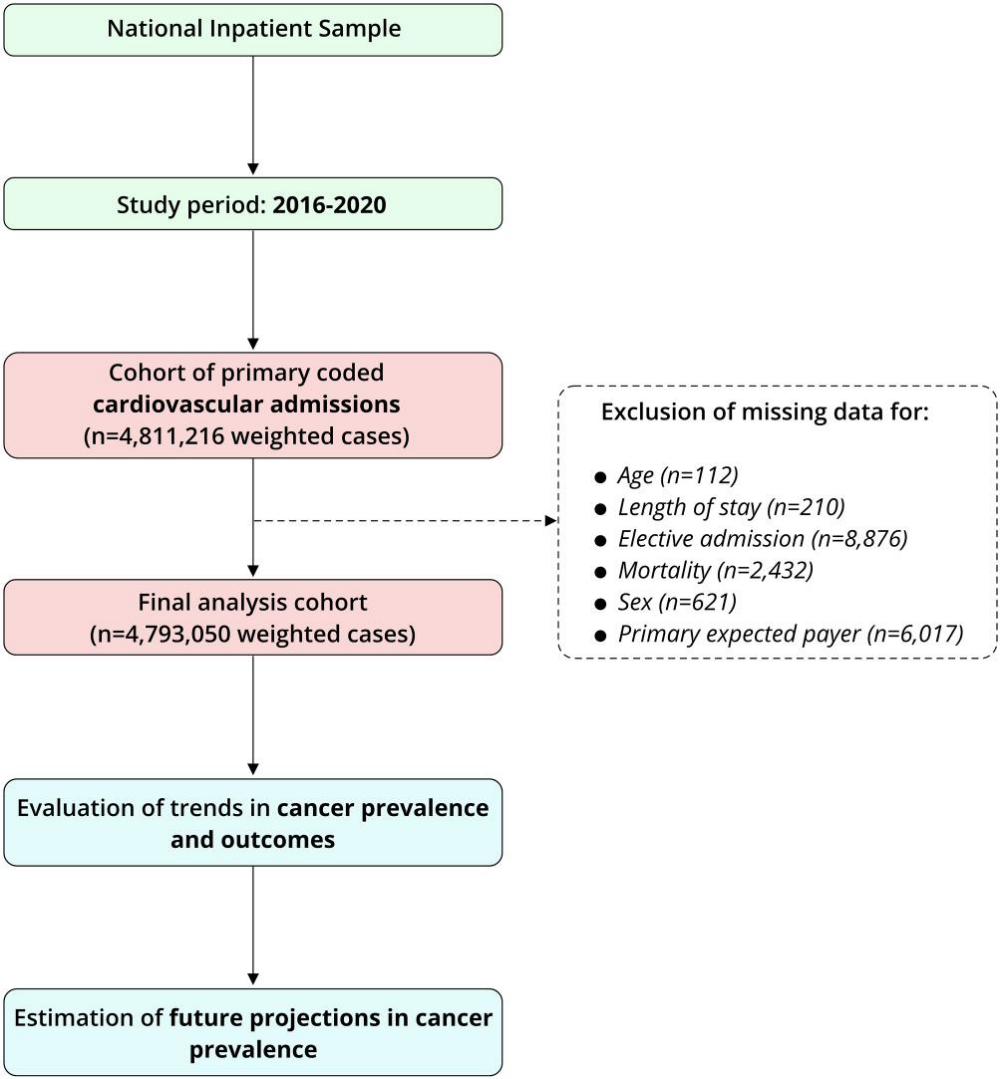
Flow diagram.

Analyses were weighted by the discharge weights provided, following HCUP recommendations.^8^ The analysis was performed after exclusion of cases due to missing data, which accounted for 0.38% (*n* = 18 268) [*Figure 1*].

### Variables—study outcomes

First, this study aimed to determine the baseline prevalence of overall and specific cancers in patients with a CV admission. Second, the analysis included an assessment of demographic and clinical characteristics of cancer patients across the study period. Finally, using the baseline 5-year period, we aimed to establish future projections in the prevalence of all cause cancer and specific cancer types among different CV admissions over the next 20 years.

### Statistical analysis

The statistical analyses were performed using Statistical Package for the Social Sciences (SPSS, version 29; IBM Corp, Armonk, NY) and Stata (version MP17, StataCorp, College Station, TX). Descriptive statistics were used to summarize patient demographic and clinical characteristics. Data were expressed as frequencies and percentages for categorical data and median (interquartile range) for continuous data. The Chi-squared test was used to compare categorical data across the years, whilst the Kruskal–Wallis test was used to compare continuous data. The Mantel–Haenszel test was used to assess linear temporal trends in the prevalence of cancer among CV admissions and in the prevalence of specific CV admissions between 2016 and 2020.

To estimate the future burden of cancer among patients hospitalized with a primary CV diagnosis, a predictive model was created. The models were based on data from 2016 to 2020. Predictions were made up to the year 2040. Different cancer subtypes were analysed separately, allowing independent estimation of trajectories for each malignancy. For each cancer group, a Poisson regression model was fitted with calendar year as a continuous predictor. Moreover, an exposure offset was incorporated into each model, to ensure relative incidence rates over time, instead of raw counts. Goodness-of-fit of the model was evaluated through the log-likelihood and pseudo *R*^2^, and standard errors were derived with the delta method. The projections were summarized using model-based incidence rate ratios (IRR) to describe relative temporal trends, along with 95% confidence intervals, using the delta method, which provides valid uncertainty quantification under standard maximum-likelihood theory. Statistical significance was defined by a *P*-value of <0.05.

## Results

### Baseline patient characteristics of cancer patients with a primary CV admission

The median age of the cohort ranged from 72 years in 2016 (IQR: 64, 81) to 73 years in 2020 (IQR: 64, 81), and there were fewer female patients within the cohort (44.0% in 2016 and 43.3% in 2020). Most patients had Caucasian ethnicity (75.7% in 2016 and 75.1% in 2020) [*Table 1*].

**Table 1:** Baseline patient characteristics of patients with cancer undergoing cardiovascular admissions across study period

Characteristics	Years					*P*-value
	2016	2017	2018	2019	2020	
Primary CV admissions with cancer	44 063	46 732	48 530	51 579	45 512	<0.001
Age (years), median (IQR)	72 (64, 81)	73 (64, 81)	73 (64, 81)	73 (65, 81)	73 (64, 81)	<0.001
Female sex, %	44.0	43.7	43.7	43.1	43.3	0.03
Weekend admission, %	20.9	21.2	21.7	21.6	20.6	<0.001
Primary expected payer, %						<0.001
Medicare	72.0	72.9	73.2	73.1	71.7	
Medicaid	6.8	6.8	6.5	6.6	6.8	
Private Insurance	17.9	17.2	16.9	16.7	17.5	
Self-pay	1.3	1.1	1.3	1.4	1.4	
No charge	0.1	0.1	0.1	0.1	0.1	
Other	2.0	1.9	2.0	2.2	2.6	
Race, %						<0.001
White	75.7	75.1	74.6	74.7	75.1	
Black	13.9	14.1	14.2	14.3	14.2	
Hispanic	5.8	6.0	6.5	6.1	5.9	
Other	4.6	4.8	4.7	4.9	4.8	
Median Household Income (percentile), %						<0.001
0–25th	27.7	26.9	26.0	27.3	26.6	
26th–50th	25.1	26.4	27.1	24.7	27.2	
51st–75th	24.7	24.3	24.7	25.5	23.8	
76th–100th	22.5	22.4	22.1	22.5	22.4	
Homelessness, %	0.2	0.2	0.3	0.3	0.3	<0.001
Cardiogenic shock, %	1.5	1.8	1.9	2.0	2.3	<0.001
Cardiac arrest, %	1.7	1.7	1.8	1.7	1.8	0.305
Do-not-resuscitate, %	16.9	18.0	18.8	19.0	20.0	<0.001
Comorbidities, %						
Chronic pulmonary disease	25.7	25.9	26.2	26.5	25.5	0.002
Chronic pancreatitis	0.2	0.3	0.2	0.2	0.3	0.017
Peptic ulcer disease	1.0	1.1	0.9	1.0	1.1	0.140
Inflammatory bowel disease	0.5	0.6	0.5	0.6	0.6	0.023
Weight loss	9.7	11.2	13.1	13.5	13.3	<0.001
HIV status	0.3	0.3	0.3	0.3	0.3	0.801
Neurologic disorders	6.6	6.9	5.8	5.8	6.0	<0.001
Psychoses	1.7	1.6	0.7	0.6	0.7	<0.001
Atrial fibrillation/flutter	35.9	36.3	37.2	37.3	35.9	<0.001
Dyslipidaemia	45.7	46.8	48.4	50.6	52.5	<0.001
Smoking	10.4	10.2	10.4	10.5	10.6	0.345
Previous stroke	8.1	8.2	8.3	8.6	8.9	<0.001
Previous AMI, PCI or CABG	21.0	20.9	20.9	20.9	20.0	<0.001
Anaemias	37.6	37.6	37.7	37.6	38.4	0.040
Fluid and electrolyte disorders	32.9	33.8	35.6	36.3	39.6	<0.001
Hypertension	74.0	76.2	77.2	78.5	78.8	<0.001
Peripheral artery disease	11.0	11.5	10.6	10.9	11.3	<0.001
Diabetes mellitus	18.2	13.0	12.2	11.6	11.0	<0.001
Coagulopathy	12.5	12.9	12.6	12.5	14.1	<0.001
Depression	10.9	11.0	11.3	11.5	11.7	<0.001
Dementia	5.7	5.9	5.6	5.5	5.8	0.060
Liver disease	4.0	4.3	4.5	4.8	5.2	<0.001
Chronic renal failure	27.4	28.6	29.6	31.1	31.2	<0.001
Valvular disease	3.2	3.0	2.7	2.8	2.7	<0.001
Alcohol or drug abuse	3.6	3.9	4.0	4.2	4.6	<0.001
Obesity	12.1	13.1	13.5	13.7	15.2	<0.001
Hypothyroidism	15.7	15.6	16.2	16.4	17.2	<0.001
Bed size of hospital, %						<0.001
Small	18.0	17.1	18.2	19.1	19.5	
Medium	28.2	28.3	28.4	28.3	27.1	
Large	53.9	54.6	53.4	52.5	53.4	
Location/teaching status of hospital, %						<0.001
Metropolitan non-teaching	21.0	22.3	20.3	17.6	17.4	
Metropolitan teaching	70.1	69.6	71.8	74.6	74.9	
Non-metropolitan hospital	8.1	8.2	7.9	7.7	7.7	
Hospitalization-related factors, median (IQR)						
Length of stay (days)	4 (2, 7)	4 (2, 7)	4 (2, 7)	4 (2, 7)	4 (2, 7)	0.171
Total charges (USD)	36 508 (19,603, 72,130)	38 550 (20,765, 76,048)	41 030 (22,103, 81,139)	43 754 (23,687, 86,778)	47 900 (25,801, 95,408)	<0.001

All analyses were weighted using the provided discharge weights as recommended by HCUP. The variables ‘HOSP_NIS’ and ‘NIS_Stratum’ were used for clustering and stratification of the data, respectively.

**Abbreviations:** CV—Cardiovscular; AIDS—Acquired Immuno-deficiency Syndrome; IQR—Interquartile Range; USD—United States Dollar.

Most patients had prevalent hypertension, with a statistically significant increase from 74.0% in 2016 to 78.8% in 2020 (*P* < 0.001). Dyslipidaemia was the second most prevalent, with a rise from 45.7% in 2016 to 52.5% in 2020 (*P* < 0.001). Prevalence of anaemias was also high, with a significant variation in frequency across the study period (37.6% in 2016 to 38.4% in 2020; *P* = 0.040). Atrial fibrillation/flutter remained relatively constant over time, with a prevalence of 35.9% in 2016, reaching a peak of 37.3% in 2019, and then going back to 35.9% in 2020 (*P* < 0.001). Chronic renal failure increased from 27.4% in 2016 to 31.2% in 2020 (*P* < 0.001), although some comorbidities, such as diabetes, exhibited a downward trend in prevalence over time (18.2% in 2016 and 11.0% in 2020; *P* < 0.001) [*Table 1*].

### Distribution of specific cardiovascular admissions in the sample

**Figure 2:**
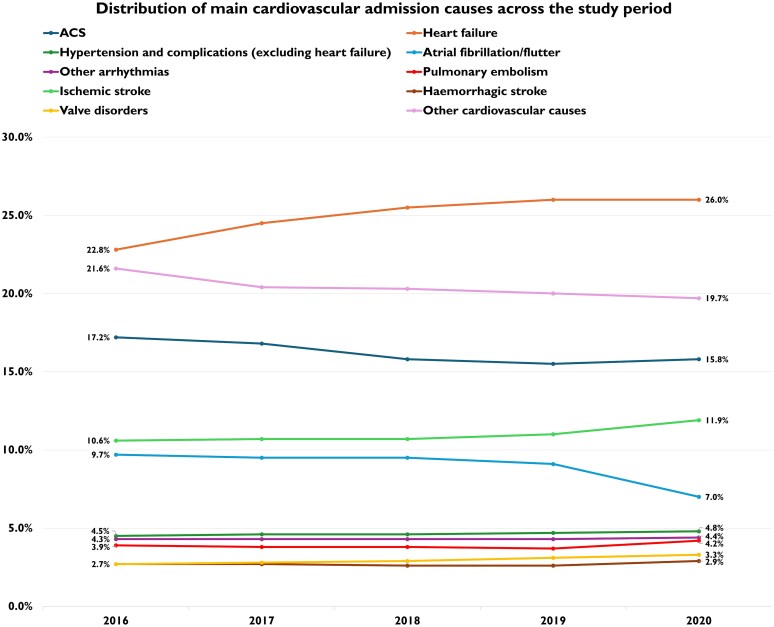
Distribution of main cardiovascular admission causes across the study period.

Considering the total study population (both patients with cancer and without cancer), the most prevalent CV admission across the study period was HF, with a significant increase from 2016 (22.8%) to 2020 (26.0%), followed by ACS (17.2% in 2016, 15.8% in 2020) and ischaemic stroke (10.6% in 2016, 11.9% in 2020). Less frequent were atrial fibrillation/flutter (9.7% in 2016, 7.0% in 2020), other arrhythmias (4.3% in 2016, 4.4% in 2020), hypertension and complications (4.5% in 2016, 4.8% in 2020), PE (3.9% in 2016, 4.2% in 2020), haemorrhagic stroke (2.7% in 2016, 3.3% in 2020) and valve disorders (2.7% in 2016, 3.3% in 2020). ACS and atrial fibrillation/flutter both showed a decreasing trend across the 5-year period [Supplementary material online, *Table S4* and *Figure 2*].

### The prevalence of cancer among patients with acute cardiovascular admissions (2016–2020)

**Figure 3:**
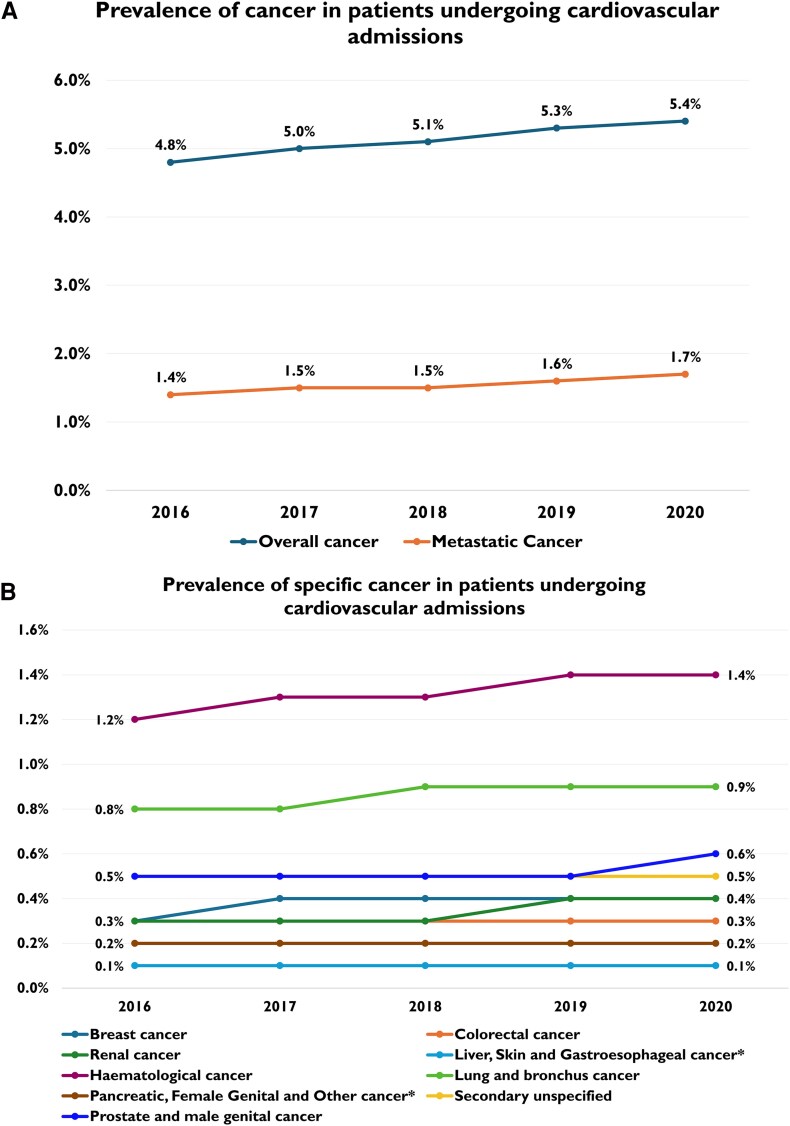
**(** *A* **)** Prevalence of overall cancer and metastatic cancer in patients undergoing cardiovascular admissions across the study period. **(***B***)** Prevalence of specific cancer in patients undergoing cardiovascular admissions across the study period. *Cancer subtypes with equal prevalence trajectories are represented by a single line.

The study included 4 793 050 patients hospitalized with a primary CV diagnosis, between 2016 and 2020. The prevalence of cancer increased significantly from 4.8% in 2016 to 5.4% in 2020 (*P* < 0.001) [*Table 2* and *Figure 3A*]. Among patients admitted with an acute CV event, 1.4% had metastatic cancer in 2016, increasing to 1.7% in 2020 (*P* < 0.001) [*Table 2*, *Figure 3A* and Supplementary material online, *Table S2*]. In relative terms, the proportion of metastatic cases among all cancers increased from 31.1% in 2016 to 32.1% in 2020 [Supplementary material online, *Table S3*].

**Table 2:** Prevalence of cancer in patients undergoing cardiovascular admissions across study period

Prevalence	Years					*P*-value^a^
	2016	2017	2018	2019	2020	
Overall Cancer, %	4.8 (*n* = 44 063)	5.0 (*n* = 46 732)	5.1 (*n* = 48 530)	5.3 (*n* = 51 579)	5.4 (*n* = 45 512)	<0.001
Metastatic cancer, %	1.4 (*n* = 13 710)	1.5 (*n* = 14 817)	1.5 (*n* = 15 189)	1.6 (*n* = 16 230)	1.7 (*n* = 14 616)	<0.001
Specific cancer type, %						
Breast cancer	0.3	0.4	0.4	0.4	0.4	<0.001
Colorectal cancer	0.3	0.3	0.3	0.3	0.3	<0.001
Renal cancer	0.3	0.3	0.3	0.4	0.4	<0.001
Liver cancer	0.1	0.1	0.1	0.1	0.1	<0.001
Haematological cancer	1.2	1.3	1.3	1.4	1.4	<0.001
Lung and bronchus cancer	0.8	0.8	0.9	0.9	0.9	<0.001
Prostate and male genital cancer	0.5	0.5	0.5	0.5	0.6	<0.001
Pancreatic cancer	0.2	0.2	0.2	0.2	0.2	<0.001
Female genital cancer	0.2	0.2	0.2	0.2	0.2	0.008
Skin cancer	0.1	0.1	0.1	0.1	0.1	<0.001
Gastroesophageal cancer	0.1	0.1	0.1	0.1	0.1	<0.001
Secondary unspecified	0.5	0.5	0.5	0.5	0.5	<0.001
Other cancer	0.2	0.2	0.2	0.2	0.2	<0.001

^a^Mantel–Haenszel test for trend.

All analyses were weighted using the provided discharge weights as recommended by HCUP. The variables ‘HOSP_NIS’ and ‘NIS_Stratum’ were used for clustering and stratification of the data, respectively.

In general, most cancer types showed a statistically significant linear increase in prevalence across the duration of the study (*P* < 0.001), although the magnitude of change in prevalence and corresponding clinical significance varied across cancer types [*Table 2* and *Figure 3B*]. Trends in both relative and absolute proportions of metastatic and non-metastatic cancers across cancer types are also reported [Supplementary material online, *Tables S2* and *S3*]. Haematological cancer was the most prevalent across all years, ranging from 1.2% in 2016 to 1.4% in 2020.

Lung cancer was the second most prevalent, growing from 0.8% in 2016 to 0.9% in 2020. Prevalence of metastatic disease among lung cancer patients increased significantly from 0.3% in 2016 to 0.4% in 2020, with a relative proportion spanning from 39.1% in 2016 to 41.4% in 2020 (*P* = 0.023) [Supplementary material online, *Tables S2* and *S3*].

Regarding breast cancer, its prevalence rose from 0.3% in 2016 to 0.4% in 2020. Trends in the prevalence of breast cancer patients with metastatic disease showed non-significant variations across the study period [Supplementary material online, *Tables S2* and *S3*].

Even though the prevalence of colorectal cancer was substantial among patients undergoing CV admission, it remained stable over the study period, with a rate of 0.3%, while there was no significant change in colorectal cancer’s metastatic disease burden [Supplementary material online, *Tables S2* and *S3*].

A growth in prostate/male genital cancer was observed, from 0.5% in 2016 to 0.6% in 2040, with metastatic cancer cases rising from 0.1% in 2016 to 0.2% in 2020, and their relative proportions growing from 29.2% in 2016 to 31.4% in 2020 (*P* = 0.108) [Supplementary material online, *Tables S2* and *S3*].

Renal/urinary tract cancer showed a modest growth in prevalence from 0.3% in 2016 to 0.4% in 2020. Burden of metastatic cancer was steady at 0.1% during the study period, with a statistically non-significant increase in relative proportion from 25.9% in 2016 to 26.4% in 2020 (*P* = 0.828) [Supplementary material online, *Tables S2* and *S3*].

The prevalence of pancreatic and female genital cancers remained stable between 2016 and 2020, both at approximately 0.2%. Prevalence of metastatic cancer among pancreatic cancer patients was 0.1% across the 5-years period, and a larger share of pancreatic cancer cases were metastatic compared to non-metastatic, ranging from 58.7% in 2016 to 60.9% in 2020 (*P* = 0.094). Metastatic burden among female genital cancer patients was also steady at 0.1%, with a significant increase in relative proportions, from 37.8% in 2016 to 42.5% in 2020 (*P* = 0.023) [Supplementary material online, *Tables S2* and *S3*].

Liver, skin, and gastroesophageal cancers not only were less prevalent but also showed less notable temporal changes across the study period. Among these, the most relevant change in prevalence of metastatic disease can be observed for skin cancer, whose burden was constant at <0.1% in absolute terms, with a steady rise in prevalence of metastatic cases compared to non-metastatic cases, from 20.8% in 2016 to 26.5% in 2020 (*P* = 0.001) [Supplementary material online, *Tables S2* and *S3*].

### Prevalence of cancer across specific cardiovascular admissions

The analysis of overall and specific cancer prevalence by CV admission type, as detailed below, is based on data reported in *Table 3*, *Figure 4A* and *B* and Supplementary material online, *Figures S3*–S15.

**Figure 4:**
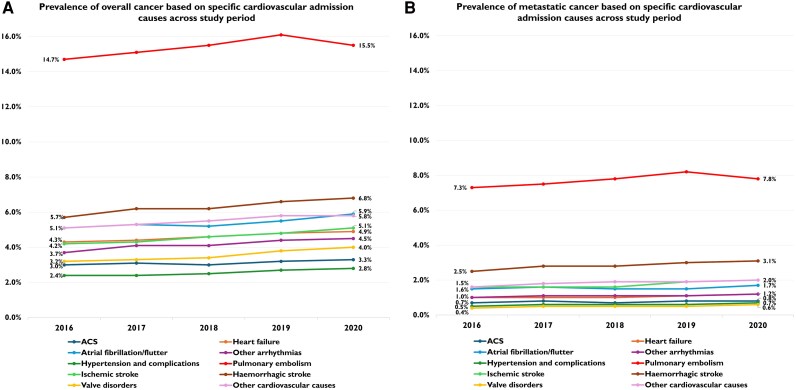
Prevalence of overall and metastatic cancer based on specific cardiovascular admission causes across study period.

**Table 3:** Prevalence of cancer based on specific cardiovascular admission causes across study period

Prevalence		Years					*P*-value
		2016	2017	2018	2019	2020	
Overall cancer, %	ACS	3.0	3.1	3	3.2	3.3	<0.001
	Heart failure	4.3	4.4	4.6	4.8	4.9	<0.001
	Atrial fibrillation/flutter	5.1	5.3	5.2	5.5	5.9	<0.001
	Other arrhythmias	3.7	4.1	4.1	4.4	4.5	<0.001
	Hypertension and complications (excluding heart failure)	2.4	2.4	2.5	2.7	2.8	<0.001
	Pulmonary embolism	14.7	15.1	15.5	16.1	15.5	<0.001
	Ischemic stroke	4.2	4.3	4.6	4.8	5.1	<0.001
	Haemorrhagic stroke	5.7	6.2	6.2	6.6	6.8	<0.001
	Valve disorders	3.2	3.3	3.4	3.8	4.0	<0.001
	Other cardiovascular causes	5.1	5.3	5.5	5.8	5.8	<0.001
Metastatic cancer, %	ACS	0.7	0.8	0.7	0.8	0.8	0.020
	Heart failure	1.0	1.0	1.0	1.1	1.2	<0.001
	Atrial fibrillation/flutter	1.5	1.6	1.5	1.5	1.7	0.001
	Other arrhythmias	1.0	1.1	1.1	1.1	1.2	0.002
	Hypertension and complications (excluding heart failure)	0.5	0.6	0.6	0.6	0.7	0.001
	Pulmonary embolism	7.3	7.5	7.8	8.2	7.8	<0.001
	Ischemic stroke	1.6	1.6	1.6	1.9	2.0	<0.001
	Haemorrhagic stroke	2.5	2.8	2.8	3.0	3.1	<0.001
	Valve disorders	0.4	0.5	0.5	0.5	0.6	0.008
	Other cardiovascular causes	1.6	1.8	1.9	1.9	2.0	<0.001
Specific cancer type, %							
Breast cancer	ACS	0.2	0.2	0.2	0.2	0.2	0.213
	Heart failure	0.3	0.3	0.4	0.4	0.4	<0.001
	Atrial fibrillation/flutter	0.4	0.4	0.4	0.4	0.5	0.181
	Other arrhythmias	0.3	0.3	0.3	0.3	0.4	<0.001
	Hypertension and complications	0.2	0.2	0.2	0.3	0.2	0.376
	Pulmonary embolism	1.3	1.4	1.3	1.4	1.3	0.865
	Ischaemic stroke	0.3	0.3	0.4	0.4	0.4	<0.001
	Haemorrhagic stroke	0.3	0.3	0.4	0.4	0.4	0.234
	Valve disorders	0.2	0.2	0.3	0.3	0.3	0.012
	Other cardiovascular causes	0.3	0.3	0.4	0.4	0.4	<0.001
Colorectal cancer	ACS	0.2	0.2	0.2	0.2	0.2	0.002
	Heart failure	0.2	0.2	0.2	0.2	0.3	<0.001
	Atrial fibrillation/flutter	0.2	0.2	0.2	0.3	0.3	0.042
	Other arrhythmias	0.2	0.2	0.2	0.2	0.2	0.275
	Hypertension and complications	0.1	0.1	0.1	0.1	0.1	0.934
	Pulmonary embolism	1.1	1.1	1.1	1.2	1.2	0.671
	Ischaemic stroke	0.2	0.2	0.2	0.3	0.3	0.005
	Haemorrhagic stroke	0.1	0.2	0.2	0.2	0.2	0.248
	Valve disorders	0.1	0.1	0.2	0.2	0.2	0.154
	Other Cardiovascular causes	0.4	0.4	0.4	0.4	0.4	0.887
Renal cancer	ACS	0.2	0.2	0.2	0.3	0.3	0.050
	Heart failure	0.3	0.3	0.3	0.4	0.3	<0.001
	Atrial Fibrillation/flutter	0.3	0.3	0.3	0.3	0.3	0.002
	Other arrhythmias	0.2	0.2	0.2	0.3	0.3	0.001
	Hypertension and complications	0.3	0.3	0.3	0.3	0.3	0.777
	Pulmonary embolism	0.7	0.8	0.8	0.8	0.9	0.310
	Ischaemic stroke	0.3	0.2	0.3	0.2	0.3	0.122
	Haemorrhagic stroke	0.3	0.3	0.3	0.4	0.3	0.232
	Valve disorders	0.2	0.2	0.3	0.3	0.3	0.036
	Other cardiovascular causes	0.3	0.4	0.4	0.4	0.4	<0.001
Liver cancer	ACS	0.1	0.1	0.1	0.1	0.1	0.536
	Heart failure	0.1	0.1	0.1	0.1	0.1	<0.001
	Atrial fibrillation/flutter	0.1	0.1	0.1	0.1	0.1	0.154
	Other arrhythmias	0.1	0.1	0.1	0.1	0.1	0.508
	Hypertension and complications	0.1	0.1	0.1	0.1	0.1	0.736
	Pulmonary embolism	0.3	0.3	0.3	0.4	0.3	0.219
	Ischaemic stroke	0.1	0.1	0.1	0.1	0.1	0.001
	Haemorrhagic stroke	0.2	0.2	0.2	0.1	0.2	0.995
	Valve disorders	0.1	0.1	0.1	0.1	0.1	0.819
	Other cardiovascular causes	0.2	0.2	0.2	0.2	0.2	0.015
Haematological cancer	ACS	0.9	0.9	0.9	0.9	0.9	0.249
	Heart failure	1.6	1.6	1.6	1.7	1.7	0.003
	Atrial fibrillation/flutter	1.4	1.4	1.4	1.5	1.6	0.017
	Other arrhythmias	1.1	1.1	1.2	1.2	1.3	0.037
	Hypertension and complications	0.7	0.7	0.7	0.8	0.8	0.584
	Pulmonary embolism	1.8	1.9	1.9	2.0	1.9	0.095
	Ischaemic stroke	0.9	0.9	1.0	1.0	1.0	0.007
	Haemorrhagic stroke	1.3	1.5	1.3	1.5	1.6	0.004
	Valve disorders	1.3	1.3	1.3	1.4	1.5	0.035
	Other cardiovascular causes	1.1	1.2	1.2	1.2	1.3	<0.001
Lung cancer	ACS	0.5	0.5	0.5	0.5	0.5	0.540
	Heart failure	0.6	0.6	0.7	0.7	0.7	<0.001
	Atrial fibrillation/flutter	1.2	1.2	1.2	1.2	1.3	0.062
	Other arrhythmias	0.6	0.6	0.6	0.6	0.6	0.605
	Hypertension and complications	0.2	0.2	0.2	0.2	0.2	0.167
	Pulmonary embolism	3.4	3.4	3.4	3.6	3.4	0.462
	Ischaemic stroke	0.8	0.8	0.9	0.9	1.0	<0.001
	Haemorrhagic stroke	0.8	1.0	0.8	0.9	0.9	0.323
	Valve disorders	0.3	0.3	0.3	0.3	0.4	0.046
	Other cardiovascular causes	0.8	0.8	0.8	0.9	0.9	0.009
Prostate and male genital cancer	ACS	0.4	0.4	0.4	0.5	0.5	<0.001
	Heart failure	0.5	0.5	0.5	0.5	0.5	0.137
	Atrial fibrillation/flutter	0.5	0.5	0.5	0.5	0.6	0.009
	Other arrhythmias	0.5	0.6	0.6	0.6	0.6	0.029
	Hypertension and complications	0.2	0.3	0.3	0.3	0.3	0.030
	Pulmonary embolism	0.8	0.9	0.8	0.9	0.9	0.184
	Ischaemic stroke	0.4	0.4	0.4	0.5	0.5	0.050
	Haemorrhagic stroke	0.5	0.5	0.6	0.5	0.7	0.018
	Valve disorders	0.5	0.5	0.5	0.6	0.6	0.160
	Other cardiovascular causes	0.5	0.5	0.5	0.6	0.6	<0.001
Pancreatic cancer	ACS	0.1	0.1	0.1	0.1	0.1	0.903
	Heart failure	0.1	0.1	0.1	0.1	0.1	0.205
	Atrial fibrillation/flutter	0.1	0.1	0.1	0.1	0.1	0.796
	Other arrhythmias	0.1	0.1	0.1	0.1	0.1	0.668
	Hypertension and complications	<0.1	<0.1	<0.1	0.1	0.1	0.140
	Pulmonary embolism	0.8	0.9	1.0	1.0	0.9	0.016
	Ischaemic stroke	0.2	0.2	0.3	0.3	0.3	<0.001
	Haemorrhagic stroke	0.1	0.1	0.1	0.1	0.2	0.409
	Valve disorders	<0.1	<0.1	<0.1	0.1	<0.1	0.166
	Other cardiovascular causes	0.2	0.2	0.2	0.2	0.3	0.021
Female genital cancer	ACS	0.1	0.1	0.1	0.1	0.1	0.756
	Heart failure	0.1	0.1	0.1	0.1	0.1	0.194
	Atrial fibrillation/flutter	0.1	0.1	0.2	0.2	0.2	0.062
	Other arrhythmias	0.1	0.1	0.1	0.1	0.1	0.725
	Hypertension and complications	0.1	0.1	0.2	0.1	0.2	0.259
	Pulmonary embolism	1.1	1.1	1.0	1.1	1.1	0.711
	Ischaemic stroke	0.2	0.2	0.2	0.2	0.2	0.468
	Haemorrhagic stroke	0.1	0.1	0.1	0.1	0.2	0.544
	Valve disorders	0.1	0.1	0.1	0.1	0.1	0.616
	Other cardiovascular causes	0.3	0.3	0.3	0.3	0.3	0.428
Skin cancer	ACS	0.1	0.1	0.1	0.1	0.1	0.873
	Heart failure	0.1	0.1	0.1	0.1	0.1	0.243
	Atrial fibrillation/flutter	0.1	0.1	0.1	0.2	0.2	0.017
	Other arrhythmias	0.1	0.1	0.1	0.2	0.1	0.113
	Hypertension and complications	0.1	0.1	0.1	0.1	0.1	0.079
	Pulmonary embolism	0.2	0.2	0.3	0.2	0.3	0.007
	Ischaemic stroke	0.1	0.1	0.1	0.1	0.1	0.063
	Haemorrhagic stroke	0.3	0.2	0.3	0.3	0.3	0.071
	Valve disorders	0.1	0.1	0.1	0.1	0.1	0.786
	Other cardiovascular causes	0.1	0.1	0.1	0.1	0.1	0.113
Gastroesophageal cancer	ACS	0.1	0.1	<0.1	0.1	0.1	0.495
	Heart failure	0.1	0.1	0.1	0.1	0.1	0.908
	Atrial fibrillation/flutter	0.1	0.1	0.1	0.1	0.2	0.321
	Other arrhythmias	0.1	0.1	0.1	0.1	0.1	0.023
	Hypertension and complications	<0.1	<0.1	<0.1	<0.1	<0.1	0.720
	Pulmonary embolism	0.4	0.4	0.5	0.5	0.5	0.028
	Ischaemic stroke	0.1	0.1	0.1	0.1	0.1	0.006
	Haemorrhagic stroke	0.1	<0.1	<0.1	0.1	0.1	0.192
	Valve disorders	<0.1	<0.1	<0.1	<0.1	<0.1	0.465
	Other cardiovascular causes	0.1	0.1	0.1	0.1	0.1	0.649
Secondary unspecified	ACS	0.2	0.3	0.2	0.3	0.3	0.006
	Heart failure	0.3	0.4	0.4	0.4	0.4	<0.001
	Atrial fibrillation/flutter	0.4	0.5	0.4	0.5	0.5	<0.001
	Other arrhythmias	0.3	0.4	0.4	0.4	0.3	0.071
	Hypertension and complications	0.2	0.2	0.2	0.2	0.3	0.036
	Pulmonary embolism	1.9	2.0	2.2	2.1	2.0	<0.001
	Ischaemic stroke	0.5	0.6	0.5	0.5	0.6	<0.001
	Haemorrhagic stroke	1.0	1.1	1.2	1.2	1.1	0.001
	Valve disorders	0.2	0.2	0.2	0.2	0.2	0.062
	Other cardiovascular causes	0.6	0.6	0.6	0.6	0.6	<0.001
Other cancer	ACS	0.1	0.1	0.1	0.1	0.1	0.090
	Heart failure	0.1	0.1	0.1	0.1	0.1	<0.001
	Atrial fibrillation/flutter	0.2	0.2	0.2	0.2	0.2	0.349
	Other arrhythmias	0.1	0.2	0.2	0.2	0.2	0.020
	Hypertension and complications	0.1	0.1	0.1	0.1	0.1	0.854
	Pulmonary embolism	0.8	0.9	0.9	0.8	0.8	0.121
	Ischaemic stroke	0.2	0.2	0.2	0.2	0.2	0.043
	Haemorrhagic stroke	0.7	0.7	0.6	0.7	0.7	0.686
	Valve disorders	<0.1	0.1	0.1	0.1	0.1	0.003
	Other cardiovascular causes	0.3	0.3	0.3	0.3	0.3	<0.001

All analyses were weighted using the provided discharge weights as recommended by HCUP. The variables ‘HOSP_NIS’ and ‘NIS_Stratum’ were used for clustering and stratification of the data, respectively.

**Abbreviations:** AIDS, Acquired Immuno-deficiency Syndrome; IQR, interquartile range; USD, United States Dollar.

#### Overall cancer

In general, the prevalence of overall cancer showed significant variations between 2016 and 2020 for all CV admissions analysed (*P* < 0.001), and its prevalence increased across all CV admission types. Likewise, the proportion of cancers with metastatic disease was observed to increase across all CV admissions from 2016 to 2020 (*P* < 0.05). Overall, cancer was most prevalent in patients with PE, significantly increasing from 14.7% in 2016 to 15.5% in 2020. Cancer was also very prevalent in patients presenting with haemorrhagic stroke, with a steady increase from 5.7% in 2016 to 6.8% in 2020. Atrial Fibrillation/flutter was the third most common CV admission in cancer patients; in this case, the rate of cancer patients increased from 5.1% in 2016 to 5.9% in 2020. Similarly, cancer prevalence also showed an increasing trend in other cardiovascular admissions, both in overall and metastatic cases, as shown in *Table 3* and *Figure 4A* and *B*.

Similar prevalence analysis was also performed for each cancer type among specific cardiovascular admissions across the study period [*Table 3* and Supplementary material online, *Figures S3*–S15]. Haematological cancer was the most common across all CV admissions, except for PE, for which lung cancer showed the highest prevalence. Moreover, haematological cancer showed the most significant increase in prevalence in patients hospitalized for HF, atrial fibrillation/flutter, ischemic and haemorrhagic stroke, and valve disorders. Lung cancer showed a notable increase in frequency in patients with HF, ischaemic stroke, and valve disorders. Other relevant findings are shown in *Table 3* and Supplementary material online, *Figures S3*–S15.

### Future projections through 2040

**Figure 5:**
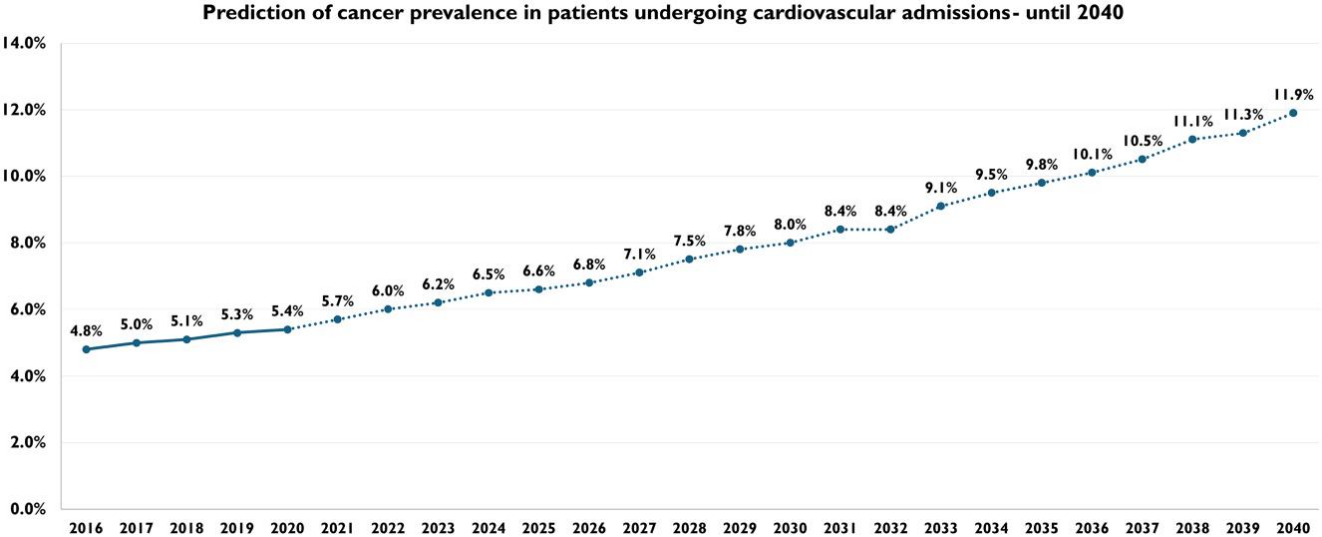
Prediction of cancer prevalence in patients undergoing cardiovascular admissions—until 2040. The solid line represents baseline data, while the dotted line represents projected data.

**Figure 6:**
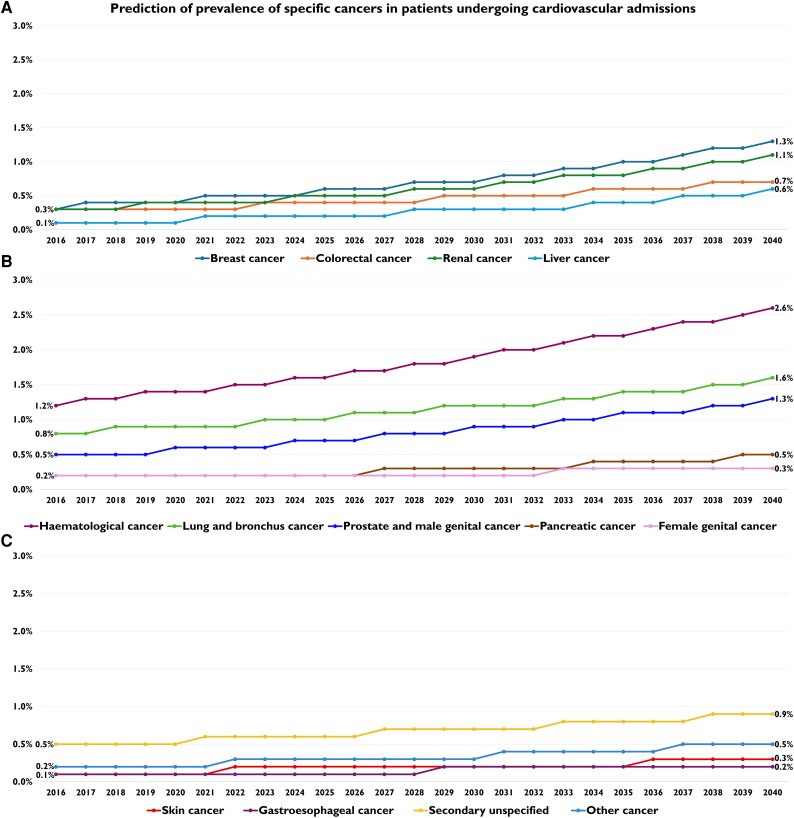
Prediction of prevalence of specific cancers in patients undergoing cardiovascular admissions—until 2040. (*A*) Breast, colorectal, renal, and liver cancer; (*B*) Haematological, lung, prostate and male genital, pancreatic, and female genital cancer; (*C*) Skin, gastroesophageal, secondary unspecified, and other cancers.

When looking at the trends in the following 20 years, until 2040, the prevalence of cancer in CV admissions is expected to nearly triple by 2040, starting from a rate of 4.8% in 2016 and reaching an estimated 11.9% in 2040 (2.48-fold increase from baseline) [Supplementary material online, *Table S5* and *Figure 5*]. The modelled projections show consistent linear increases across all major cancer types [Supplementary material online, *Table S5*, Supplementary material online, *Tables S6A*-*D*, and *Figure 6A–C*, Supplementary material online, *Figures S1A*-*F* and *S2A*-*G*]. While haematological and lung cancers are expected to remain the cancers with the highest total burden, other malignancies show faster growth trajectories, with bigger relative growth. Liver cancer showed the steepest annual increase in prevalence, from 0.1% in 2016 to 0.6% in 2040 (IRR 1.069, 95% CI 1.049–1.089; *P* < 0.001) [Supplementary material online, *Figure S1D*], followed by breast cancer, which is expected to increase from 0.3% in 2016 to 1.3% in 2040 (IRR 1.056, 95% CI 1.045–1.068; *P* < 0.001) [Supplementary material online, *Figure S1A*], and renal cancer, which showed a future increasing trend from 0.3% in 2016 to 1.1% in 2040 (IRR 1.055, 95% CI 1.043–1.067; *P* < 0.001) [Supplementary material online, *Figure S1C*]. Pancreatic and colorectal cancers are also expected to more than double in prevalence, with the former that is expected to rise from 0.2% in 2016 to 0.5% in 2040 (IRR 1.049, 95% CI 1.032–1.066; *P* < 0.001) [Supplementary material online, *Figure S2B*], while the latter is projected to rise from 0.3% in 2016 to 0.7% in 2040 (IRR 1.044, 95% CI 1.031–1.056; *P* < 0.001) [Supplementary material online, *Figure S1B*]. Skin cancer shows similar trends, tripling from a rate of 0.1% in 2016 to 0.3% in 2040 (IRR 1.038, 95% CI 1.019–1.057; *P* < 0.001) [Supplementary material online, *Figure S2D*]. Prostate and male genital cancer are predicted to show a quite linear and constant increase across the years, from 0.5% in 2016 to 1.3% in 2040 (IRR 1.042, 95% CI 1.033–1.052; *P* < 0.001) [Supplementary material online, *Figure S2A*]. Similarly, haematological cancer is projected to rise from 1.2% in 2016 to 2.6% in 2040 (IRR 1.032, 95% CI 1.026–1.037; *P* < 0.001) [Supplementary material online, *Figure S1E*]. Lung and bronchus cancer is projected to double from 0.8% in 2016 to 1.6% in 2040 (IRR 1.028, 95% CI 1.021–1.035; *P* < 0.001) [Supplementary material online, *Figure S1F*]. Comparable findings are evident in case of gastroesophageal cancer, with a growth rate ranging from 0.1% in 2016, expected to rise to 0.2% in 2040 (IRR 1.038, 95% CI 1.017–1.060; *P* < 0.001) [Supplementary material online, *Figure S2E*]. Female genital cancer has a less relevant rate of increase until 2040, increasing from 0.2% in 2016 to 0.3% in 2040 (IRR 1.017, 95% CI 1.015–1.032; *P* = 0.031) [Supplementary material online, *Figure S2C*]. In terms of absolute changes, haematological cancer is expected to remain the most common across the years and is predicted to show the highest absolute increase (+1.4% absolute increase), followed by breast cancer (+1.0% absolute increase) and lung and bronchus cancer (+0.8% absolute increase). These represent the cancers with a highest total burden.

## Discussion

This nationwide analysis of US hospitalized patients with primary CV diagnosis and co-existing cancer adds important data on contemporary status and future projections of cancer in this high-risk population. The study revealed a steady rise in cancer prevalence in the period from 2016 to 2020. Consistently, the present projections indicate a continuous increase in the cancer prevalence among CV inpatients, meaning that by 2040, roughly one out of eight patients admitted for a CV condition may also carry a diagnosis of concurrent cancer. Importantly, the magnitude of these projections should be interpreted with caution and viewed as exploratory and illustrative of potential population-level trajectories. These findings emphasize a growing co-existence of cancer and CV disease. This is in line with the improvement in both CVD and cancer treatment, leading to prolonged survival in both cohorts, and with the presence of a rising ageing population on a global scale,^9^ which greatly contributes to the growing prevalence of multimorbidity,^10^ and represents a major risk factor for many CV and cancer pathologies.^11,12^ Consistent with the population-level trends observed in our study, Global Burden of Disease (GBD) forecasts indicate that the absolute (crude) burden of disability-adjusted life years (DALYs) attributable to CVD and cancer has increased from 1990 to 2021, and is projected to rise further by 2050, largely driven by population ageing and growth. In contrast, age-standardized DALY rates attributable to these conditions have declined and are expected to keep decreasing by 2050, which is reflective of improved preventive and treatment measures.^13^

To the best of our knowledge, this is the first national analysis of future cancer projections in patients admitted with an acute CV event. Among the few published studies on this topic, Okura et al. analysed a local Japanese cancer registry and estimated a 23.4% increase in cancer patients with co-existing CV disease by 2039, with a growth rate (1.23) outpacing the cancer-only population (1.18). Atrial fibrillation was predicted to be the most common CV comorbidity, with a more pronounced growth in females, though absolute numbers remained higher in males.^14^

Several mechanisms may explain the rising cancer prevalence among patients with CV disease. First, a shared risk hypothesis suggests that increasing rates of common risk factors and comorbidities contribute to both cancer and CVD. These include smoking, obesity, type 2 diabetes, hypertension, metabolic syndrome, sedentary lifestyle, and poor diet. Obesity and diabetes are strongly linked to several cancer types and are well-established drivers of CVD, including coronary artery disease, stroke, and HF.^15^ The World Obesity Atlas (2025) projects that about three billion adults will be overweight by 2030, with 1.1 billion classified as obese and more prevalent among women (643 million).^16^ The prevalence of diabetes is also predicted to rise to 43% and affect approximately 1 in 8 adults worldwide (∼853 million) by 2050.^17^ Smoking is causally implicated in the pathogenesis of several cancers, especially lung cancer, whose risk is 15- to 30-fold higher in smokers,^18^ and is also linked to an elevated risk of CVD.^19^ While smoking rates are projected to decline by 25.9% in males and 30.0% in females by 2050, total numbers will remain substantial.^20^ According to GBD forecasts, high systolic blood pressure, dietary risks and air pollution are projected to be the leading risk factors driving the shared burden of CVD and cancer in 2050.^13^ Inflammation underlies many of these risks, promoting both tumorigenesis and atherogenesis. Chronic inflammation supports tumour growth by encouraging cellular transformation, reactive oxygen, tissue damage, and angiogenesis dysfunction.^21^ Pro-inflammatory cytokines like IL-6, TNF-α and IL-1β activate pathways that promote tumour proliferation, angiogenesis, and apoptosis resistance.^22^ Similarly, inflammation promotes atherogenesis through immune cell recruitment, endothelial dysfunction, and atherosclerosis, promoting plaque growth and disruption.^23^

Second, increased survival with better treatment modalities, as well as the rising issue of cardiotoxicity could greatly contribute to increased prevalence. It is well established that many cancer drugs are associated with an increased risk of CV disease, such as left ventricular dysfunction, HF, hypertension, myocardial ischaemia, and rhythm abnormalities.^24^ A US-based study predicts an increase in the proportion of cancer survivors, from an estimated 18.1 million in 2022 to 26 million by 2040.^25^ Notably, recent studies confirm there is an overall ∼42% higher risk of CVD in cancer survivors than people without cancer, and that survivors of breast, lung, and haematological cancers experience some of the highest rates of CV adverse events.^26^ Observational studies confirm that cancer survivors are at increased risk of hospitalization (HR 1.11) and/or death from CVD (HR 1.31) compared to individuals without a history of cancer, especially survivors of lung cancer, multiple myeloma, and non-Hodgkin lymphoma and breast cancer.^27^ Another study highlighted a 55% higher risk of mortality from CVD in cancer survivors compared to the general population.^4^

Third, another important factor contributing to the increasing prevalence of cancer in the general population, and consequently among those with CV admissions, is the continuous trend towards population ageing. This demographic shift is expected to significantly raise cancer incidence in the next decades. This is supported by the International Agency for Research on Cancer (IARC), which estimated a projected rise of new cancer cases from 19.3 million in 2020 to 28.4 million in 2040, which corresponds to a 47% increase.^28^ Even more recent estimates suggest an increase to over 35 million annual cases by 2050, corresponding to a 77% increase from the 20 million cases in 2022.^29^ Projections suggest that breast, prostate, lung, and colorectal cancers will remain the most frequent, with female breast cancer predicted to rise by about 52% by 2050 due to demographic changes.^30^ These trends align with our findings, as higher cancer prevalence will be associated with increasing CV complications.

When looking at the specific cancers, liver, breast, renal, and pancreatic cancer showed the highest relative increase in prevalence. This trend is supported by epidemiological projections indicating a significant rise in both liver and pancreatic cancers worldwide by 2040. In particular, liver cancer, which was the one with the overall steepest relative projected increase in patients with CV admissions, is expected to surge dramatically in the general population, with a predicted 55% increase in incidence from 2020 to 2040.^31^

In terms of absolute increase and prevalence, haematological, breast, prostate, and lung cancers are predicted to remain the most common until 2040. Haematological cancer was the most prevalent among patients admitted with CVD. It was especially frequent in patients with PE, HF, and atrial fibrillation, likely due to shared pathogenesis and treatment-related cardiotoxicity.^32^ Breast cancer also had one of the highest predicted absolute and relative rise in prevalence among CV admissions, possibly related to cardiotoxic effects of therapies like anthracyclines and HER2-targeted agents, known to cause left ventricular dysfunction and HF, particularly when combined with anthracyclines.^33^ In addition, radiation therapy to the thorax can also affect the heart, increasing the risk of CV complications.^34^ Lung cancer also demonstrated a significant projected absolute rise and prevalence, remaining the most frequent malignancy among patients with PE, which is consistent with the literature.^35^ Among cardiotoxic treatments, immune checkpoint inhibitors have been linked to CV adverse events including myocarditis, pericardial diseases, and arrhythmias,^36^ while targeted therapies such as EGFR tyrosine kinase inhibitors, used to treat non-small-cell lung cancer, have been associated with systolic dysfunction and HF.^37^

It is important to keep in mind that our projections were derived using a Poisson Regression approach that models observed temporal trends in cancer prevalence among patients with cardiovascular disease, without explicitly incorporating individual-level determinants. This approach is well-suited for prediction when trends are stable and show no statistical evidence of non-linearity, as observed in our data. Accordingly, the projections reflect extrapolations of observed temporal trends rather than assumptions regarding future changes in the underlying determinants of cancer and CVD prevalence. Nonetheless, future deviations from the projected trends may occur if such determinants evolve over time. Such changes may include improvements in cancer prevention and early detection, improved treatments, reduced exposure to cardiotoxic agents, or advances in CV management leading to modified hospitalization patterns. Conversely, population ageing, increasing multimorbidity, and increased exposure to risk factors could accelerate the predicted trends. In this context, public health plays a fundamental role in trying to counterbalance this increase by implementing broader primary and secondary prevention strategies. Although our analysis does not evaluate clinical interventions directly, the observed and projected trends provide context for healthcare systems to anticipate a growing need for integrated care models that incorporate CV prevention into oncology pathways and survivorship care.

Managing such a fragile and growing cohort highlights the relevance of enhanced clinical education. International guidelines increasingly endorse the integrated management of these patients. European Society of Cardiology (ESC) Guidelines on cardio-oncology (2022) already suggest a multidisciplinary approach to patients developing CV complications from cancer therapy.^38^ The American Heart Association (AHA) has introduced the concept of cardio-oncology rehabilitation (CORE), which focuses on strategies to identify cancer patients or survivors at risk of CVD and to prevent or cope with such adverse events. These strategies, which adapt cardiac rehab principles to cancer patients, include counseling on exercise training and on CV risk factors management (weight, blood pressure, lipid profile, weight loss, diabetes mellitus, tobacco cessation).^39^ ESC and American Society of Clinical Oncology (ASCO) guidelines advocate for baseline CV risk stratification, using tools like the HFA-ICOS risk assessment and cardiac tests (e.g. electrocardiogram, biomarkers, and imaging) before starting potentially cardiotoxic treatments. This enables the identification of at-risk patients and to tailor primary or secondary preventive measures according to pre-existing CVD, individual risk of CV toxicity, and type of cancer therapy.^40^ In addition, these findings may help raise awareness of the potential usefulness of cancer screening programmes in patients with CVD.

Beyond clinical practice guidelines, national and international policy initiatives may play an important role in supporting the development of cardio-oncology care frameworks. Among emerging ones, at the national level, in the US the National Cancer Institute (NCI) in partnership with the National Heart, Lung, and Blood Institute (NHLBI), has established a joint cardio-oncology research initiative, which aims to advance research in cancer treatment-related cardiotoxicity and to promote collaboration between cardiologists and oncologists. Their collaboration has led to several studies, e.g. a NCI/NHLBI-supported research identified ways to mitigate cardiotoxicity, including the use of beta-blockers and dexrazoxane.^41^ Moreover, in the UKa dedicated cardio-oncology clinic opened in 2011 at the Royal Brompton Hospital. Between February 2011 and February 2016, it managed 535 patients experiencing cancer treatment-related cardiotoxicity, with 128 presenting with left ventricular systolic dysfunction (LVSD). Following cardiac optimization, LVEF improved from 45% to 53% (*P* < 0.001) and the percentage of patients considered eligible to continue cancer therapy rose to 88%.^42^ Therefore, results are promising and encourage the implementation of such clinics on a broader scale.

Finally, although this analysis is based on US inpatient administrative data, its findings may have relevance beyond the US context and require cautious interpretation when applied to European healthcare contexts. Europe bears one fifth of the global cancer cases (22.4%) and cancer deaths (20.4%), despite accounting for less than 10% of the global population,^28^ reflecting an ageing population and an improving cancer survival rate. These demographic trends, coupled with the increasing prevalence of shared CV and cancer risk factors, suggest that the observed population-level increase in cancer diagnoses among CV hospitalizations may also be directionally relevant to European settings. However, important structural differences between USA and European healthcare systems limit the direct extrapolation of absolute prevalence estimates, since European systems are largely publicly funded, with universal coverage, different hospitalization thresholds, and distinct reimbursement and coding practices that may influence admission patterns and diagnostic recording.^43,44^ Nevertheless, the broader implications remain highly relevant for Europe and other non-US countries.

## Limitations

Several limitations are present in this study, mainly related to the use of administrative data and ICD-10 coding. This includes potential for inaccuracy and inconsistency in diagnostic coding. The structure of ICD-10 coding may lead to grouping of distinct clinical entities under single categories, thereby limiting the interpretability of results within certain CV and cancer diagnoses. Moreover, the NIS does not provide detailed anthropometric, lifestyle or clinical data, such as cancer stage, cancer treatment exposure, or timing of cancer diagnosis relative to hospitalization. Cancer diagnoses are therefore not stratified by the status of the disease during CV admission, so whether the disease is active, in remission, or currently undergoing treatment. As a result, potential mechanisms linking cancer and CVD, such as cardiotoxicity of oncologic therapies, cannot be assessed, and the present analysis remains descriptive at the population-level. Furthermore, the NIS records anonymized discharge-level data, which does not allow identification of repeated hospitalizations for the same individual over time. Consequently, patients with chronic CV conditions requiring frequent admissions, such as heart failure or atrial fibrillation, may contribute to multiple records, potentially inflating prevalence estimates. This limitation may also influence long-term projections, and its magnitude cannot be fully quantified. Importantly, this study focused only on inpatients, so it is not possible to extrapolate these findings to the overall population of patients with CV conditions. Projections were derived using a Poisson model, which, although appropriate for our aims, does not account for changes in the characteristics of the population of interest and does not model linear or non-linear associations between demographic or epidemiological factors and the outcomes of interest, at person-level. The projected trends were not externally validated using independent datasets, as such analysis is not feasible within the NIS framework. The long-term extrapolations are based on a five-year observation window, which also warrants careful interpretation of projected trends. However, extending the analysis to earlier periods with different coding structures or temporal dynamics may introduce additional bias and was therefore not pursued. Additionally, as this analysis is based on aggregate administrative data, these trends reflect population-level patterns, individual-level association or causal relationships between CVD and cancer cannot be directly inferred (ecological fallacy). Finally, as these results are based on US data, they may not be easily generalizable to countries with different healthcare systems and differing cancer and CVD epidemiology. Additionally, future temporal changes in coding practices, diagnostic sensitivity, and admission thresholds may influence the predicted trends. Finally, the structure and coding system of the NIS dataset may further limit reproducibility of these findings in non-US settings. Validation of these trends in international administrative datasets would be valuable to assess generalizability. Despite these limitations, this study leverages a very large dataset, offering novel insights and a broad overview of the relationship at population-level between CV disease and cancer.

## Conclusion

This study provides new insights into future trends in the prevalence of cancer diagnoses among CV hospitalizations, forecasting a significant increase from 2016 to 2040. Specifically, the proportion of CV hospitalizations with a concurrent cancer diagnosis is projected to increase approximately from 1 in 21 in 2016 to 1 in 8 by 2040. This prediction is relevant for healthcare planning, policies, and further granular research, in the developing field of cardio-oncology.

## Supplementary Material

oeag030_Supplementary_Data

## Data Availability

The data underlying this article were provided by HCUP under license and cannot be shared publicly. Researchers may access the NIS dataset directly from https://www.hcup-us.ahrq.gov upon fulfilling the data use agreement requirements.

## References

[1] Roth GA, Abate D, Abate KH, Abay SM, Abbafati C, Abbasi N, Abbastabar H, Abd-Allah F, Abdela J, Abdelalim A, Abdollahpour I, Abdulkader RS, Abebe HT, Abebe M, Abebe Z, Abejie AN, Abera SF, Abil OZ, Abraha HN, Abrham AR, Abu-Raddad LJ, Accrombessi MMK, Acharya D, Adamu AA, Adebayo OM, Adedoyin RA, Adekanmbi V, Adetokunboh OO, Adhena BM, Adib MG, Admasie A, Afshin A, Agarwal G, Agesa KM, Agrawal A, Agrawal S, Ahmadi A, Ahmadi M, Ahmed MB, Ahmed S, Aichour AN, Aichour I, Aichour MTE, Akbari ME, Akinyemi RO, Akseer N, Al-Aly Z, Al-Eyadhy A, Al-Raddadi RM, Alahdab F, Alam K, Alam T, Alebel A, Alene KA, Alijanzadeh M, Alizadeh-Navaei R, Aljunid SM, Alkerwi A, Alla F, Allebeck P, Alonso J, Altirkawi K, Alvis-Guzman N, Amare AT, Aminde LN, Amini E, Ammar W, Amoako YA, Anber NH, Andrei CL, Androudi S, Animut MD, Anjomshoa M, Ansari H, Ansha MG, Antonio CAT, Anwari P, Aremu O, Ärnlöv J, Arora A, Arora M, Artaman A, Aryal KK, Asayesh H, Asfaw ET, Ataro Z, Atique S, Atre SR, Ausloos M, Avokpaho EFGA, Awasthi A, Quintanilla BPA, Ayele Y, Ayer R, Azzopardi PS, Babazadeh A, Bacha U, Badali H, Badawi A, Bali AG, Ballesteros KE, Banach M, Banerjee K, Bannick MS, Banoub JAM, Barboza MA, Barker-Collo SL, Bärnighausen TW, Barquera S, Barrero LH, Bassat Q, Basu S, Baune BT, Baynes HW, Bazargan-Hejazi S, Bedi N, Beghi E, Behzadifar M, Behzadifar M, Béjot Y, Bekele BB, Belachew AB, Belay E, Belay YA, Bell ML, Bello AK, Bennett DA, Bensenor IM, Berman AE, Bernabe E, Bernstein RS, Bertolacci GJ, Beuran M, Beyranvand T, Bhalla A, Bhattarai S, Bhaumik S, Bhutta ZA, Biadgo B, Biehl MH, Bijani A, Bikbov B, Bilano V, Bililign N, Bin Sayeed MS, Bisanzio D, Biswas T, Blacker BF, Basara BB, Borschmann R, Bosetti C, Bozorgmeh K, Brady OJ, Brant LC, Brayne C, Brazinova A, Breitborde NJK, Brenner H, Briant PS, Britton G, Brugha T, Busse R, Butt ZA, Callender CSKH, Campos-Nonato IR, Campuzano Rincon JC, Cano J, Car M, Cárdenas R, Carreras G, Carrero JJ, Carter A, Carvalho F, Castañeda-Orjuela CA, Castillo Rivas J, Castle CD, Castro C, Castro F, Catalá-López F, Cerin E, Chaiah Y, Chang J-C, Charlson FJ, Chaturvedi P, Chiang PP-C, Chimed-Ochir O, Chisumpa VH, Chitheer A, Chowdhury R, Christensen H, Christopher DJ, Chung S-C, Cicuttini FM, Ciobanu LG, Cirillo M, Cohen AJ, Cooper LT, Cortesi PA, Cortinovis M, Cousin E, Cowie BC, Criqui MH, Cromwell EA, Crowe CS, Crump JA, Cunningham M, Daba AK, Dadi AF, Dandona L, Dandona R, Dang AK, Dargan PI, Daryani A, Das SK, Gupta RD, Neves JD, Dasa TT, Dash AP, Davis AC, Davis Weaver N, Davitoiu DV, Davletov K, De La Hoz FP, De Neve J-W, Degefa MG, Degenhardt L, Degfie TT, Deiparine S, Demoz GT, Demtsu BB, Denova-Gutiérrez E, Deribe K, Dervenis N, Des Jarlais DC, Dessie GA, Dey S, Dharmaratne SD, Dicker D, Dinberu MT, Ding EL, Dirac MA, Djalalinia S, Dokova K, Doku DT, Donnelly CA, Dorsey ER, Doshi PP, Douwes-Schultz D, Doyle KE, Driscoll TR, Dubey M, Dubljanin E, Duken EE, Duncan BB, Duraes AR, Ebrahimi H, Ebrahimpour S, Edessa D, Edvardsson D, Eggen AE, El Bcheraoui C, El Sayed Zaki M, El-Khatib Z, Elkout H, Ellingsen CL, Endres M, Endries AY, Er B, Erskine HE, Eshrati B, Eskandarieh S, Esmaeili R, Esteghamati A, Fakhar M, Fakhim H, Faramarzi M, Fareed M, Farhadi F, Farinha CSE, Faro A, Farvid MS, Farzadfar F, Farzaei MH, Feigin VL, Feigl AB, Fentahun N, Fereshtehnejad S-M, Fernandes E, Fernandes JC, Ferrari AJ, Feyissa GT, Filip I, Finegold S, Fischer F, Fitzmaurice C, Foigt NA, Foreman KJ, Fornari C, Frank TD, Fukumoto T, Fuller JE, Fullman N, Fürst T, Furtado JM, Futran ND, Gallus S, Garcia-Basteiro AL, Garcia-Gordillo MA, Gardner WM, Gebre AK, Gebrehiwot TT, Gebremedhin AT, Gebremichael B, Gebremichael TG, Gelano TF, Geleijnse JM, Genova-Maleras R, Geramo YCD, Gething PW, Gezae KE, Ghadami MR, Ghadimi R, Ghasemi Falavarjani K, Ghasemi-Kasman M, Ghimire M, Gibney KB, Gill PS, Gill TK, Gillum RF, Ginawi IA, Giroud M, Giussani G, Goenka S, Goldberg EM, Goli S, Gómez-Dantés H, Gona PN, Gopalani SV, Gorman TM, Goto A, Goulart AC, Gnedovskaya EV, Grada A, Grosso G, Gugnani HC, Guimaraes ALS, Guo Y, Gupta PC, Gupta R, Gupta R, Gupta T, Gutiérrez RA, Gyawali B, Haagsma JA, Hafezi-Nejad N, Hagos TB, Hailegiyorgis TT, Hailu GB, Haj-Mirzaian A, Haj-Mirzaian A, Hamadeh RR, Hamidi S, Handal AJ, Hankey GJ, Harb HL, Harikrishnan S, Haro JM, Hasan M, Hassankhani H, Hassen HY, Havmoeller R, Hay RJ, Hay SI, He Y, Hedayatizadeh-Omran A, Hegazy MI, Heibati B, Heidari M, Hendrie D, Henok A, Henry NJ, Herteliu C, Heydarpour F, Heydarpour P, Heydarpour S, Hibstu DT, Hoek HW, Hole MK, Homaie Rad E, Hoogar P, Hosgood HD, Hosseini SM, Hosseinzadeh M, Hostiuc M, Hostiuc S, Hotez PJ, Hoy DG, Hsiao T, Hu G, Huang JJ, Husseini A, Hussen MM, Hutfless S, Idrisov B, Ilesanmi OS, Iqbal U, Irvani SSN, Irvine CMS, Islam N, Islam SMS, Islami F, Jacobsen KH, Jahangiry L, Jahanmehr N, Jain SK, Jakovljevic M, Jalu MT, James SL, Javanbakht M, Jayatilleke AU, Jeemon P, Jenkins KJ, Jha RP, Jha V, Johnson CO, Johnson SC, Jonas JB, Joshi A, Jozwiak JJ, Jungari SB, Jürisson M, Kabir Z, Kadel R, Kahsay A, Kalani R, Karami M, Karami Matin B, Karch A, Karema C, Karimi-Sari H, Kasaeian A, Kassa DH, Kassa GM, Kassa TD, Kassebaum NJ, Katikireddi SV, Kaul A, Kazemi Z, Karyani AK, Kazi DS, Kefale AT, Keiyoro PN, Kemp GR, Kengne AP, Keren A, Kesavachandran CN, Khader YS, Khafaei B, Khafaie MA, Khajavi A, Khalid N, Khalil IA, Khan EA, Khan MS, Khan MA, Khang Y-H, Khater MM, Khoja AT, Khosravi A, Khosravi MH, Khubchandani J, Kiadaliri AA, Kibret GD, Kidanemariam ZT, Kiirithio DN, Kim D, Kim Y-E, Kim YJ, Kimokoti RW, Kinfu Y, Kisa A, Kissimova-Skarbek K, Kivimäki M, Knudsen AKS, Kocarnik JM, Kochhar S, Kokubo Y, Kolola T, Kopec JA, Koul PA, Koyanagi A, Kravchenko MA, Krishan K, Kuate Defo B, Kucuk Bicer B, Kumar GA, Kumar M, Kumar P, Kutz MJ, Kuzin I, Kyu HH, Lad DP, Lad SD, Lafranconi A, Lal DK, Lalloo R, Lallukka T, Lam JO, Lami FH, Lansingh VC, Lansky S, Larson HJ, Latifi A, Lau KM-M, Lazarus JV, Lebedev G, Lee PH, Leigh J, Leili M, Leshargie CT, Li S, Li Y, Liang J, Lim L-L, Lim SS, Limenih MA, Linn S, Liu S, Liu Y, Lodha R, Lonsdale C, Lopez AD, Lorkowski S, Lotufo PA, Lozano R, Lunevicius R, Ma S, Macarayan ERK, Mackay MT, MacLachlan JH, Maddison ER, Madotto F, Magdy Abd El Razek H, Magdy Abd El Razek M, Maghavani DP, Majdan M, Majdzadeh R, Majeed A, Malekzadeh R, Malta DC, Manda A-L, Mandarano-Filho LG, Manguerra H, Mansournia MA, Mapoma CC, Marami D, Maravilla JC, Marcenes W, Marczak L, Marks A, Marks GB, Martinez G, Martins-Melo FR, Martopullo I, März W, Marzan MB, Masci JR, Massenburg BB, Mathur MR, Mathur P, Matzopoulos R, Maulik PK, Mazidi M, McAlinden C, McGrath JJ, McKee M, McMahon BJ, Mehata S, Mehndiratta MM, Mehrotra R, Mehta KM, Mehta V, Mekonnen TC, Melese A, Melku M, Memiah PTN, Memish ZA, Mendoza W, Mengistu DT, Mengistu G, Mensah GA, Mereta ST, Meretoja A, Meretoja TJ, Mestrovic T, Mezgebe HB, Miazgowski B, Miazgowski T, Millear AI, Miller TR, Miller-Petrie MK, Mini GK, Mirabi P, Mirarefin M, Mirica A, Mirrakhimov EM, Misganaw AT, Mitiku H, Moazen B, Mohammad KA, Mohammadi M, Mohammadifard N, Mohammed MA, Mohammed S, Mohan V, Mokdad AH, Molokhia M, Monasta L, Moradi G, Moradi-Lakeh M, Moradinazar M, Moraga P, Morawska L, Moreno Velásquez I, Morgado-Da-Costa J, Morrison SD, Moschos MM, Mouodi S, Mousavi SM, Muchie KF, Mueller UO, Mukhopadhyay S, Muller K, Mumford JE, Musa J, Musa KI, Mustafa G, Muthupandian S, Nachega JB, Nagel G, Naheed A, Nahvijou A, Naik G, Nair S, Najafi F, Naldi L, Nam HS, Nangia V, Nansseu JR, Nascimento BR, Natarajan G, Neamati N, Negoi I, Negoi RI, Neupane S, Newton CRJ, Ngalesoni FN, Ngunjiri JW, Nguyen AQ, Nguyen G, Nguyen HT, Nguyen HT, Nguyen LH, Nguyen M, Nguyen TH, Nichols E, Ningrum DNA, Nirayo YL, Nixon MR, Nolutshungu N, Nomura S, Norheim OF, Noroozi M, Norrving B, Noubiap JJ, Nouri HR, Nourollahpour Shiadeh M, Nowroozi MR, Nyasulu PS, Odell CM, Ofori-Asenso R, Ogbo FA, Oh I-H, Oladimeji O, Olagunju AT, Olivares PR, Olsen HE, Olusanya BO, Olusanya JO, Ong KL, Ong SKS, Oren E, Orpana HM, Ortiz A, Ortiz JR, Otstavnov SS, Øverland S, Owolabi MO, Özdemir R, P A M, Pacella R, Pakhale S, Pakhare AP, Pakpour AH, Pana A, Panda-Jonas S, Pandian JD, Parisi A, Park E-K, Parry CDH, Parsian H, Patel S, Pati S, Patton GC, Paturi VR, Paulson KR, Pereira A, Pereira DM, Perico N, Pesudovs K, Petzold M, Phillips MR, Piel FB, Pigott DM, Pillay JD, Pirsaheb M, Pishgar F, Polinder S, Postma MJ, Pourshams A, Poustchi H, Pujar A, Prakash S, Prasad N, Purcell CA, Qorbani M, Quintana H, Quistberg DA, Rade KW, Radfar A, Rafay A, Rafiei A, Rahim F, Rahimi K, Rahimi-Movaghar A, Rahman M, Rahman MHU, Rahman MA, Rai RK, Rajsic S, Ram U, Ranabhat CL, Ranjan P, Rao PC, Rawaf DL, Rawaf S, Razo-García C, Reddy KS, Reiner RC, Reitsma MB, Remuzzi G, Renzaho AMN, Resnikoff S, Rezaei S, Rezaeian S, Rezai MS, Riahi SM, Ribeiro ALP, Rios-Blancas MJ, Roba KT, Roberts NLS, Robinson SR, Roever L, Ronfani L, Roshandel G, Rostami A, Rothenbacher D, Roy A, Rubagotti E, Sachdev PS, Saddik B, Sadeghi E, Safari H, Safdarian M, Safi S, Safiri S, Sagar R, Sahebkar A, Sahraian MA, Salam N, Salama JS, Salamati P, Saldanha RDF, Saleem Z, Salimi Y, Salvi SS, Salz I, Sambala EZ, Samy AM, Sanabria J, Sanchez-Niño MD, Santomauro DF, Santos IS, Santos JV, Milicevic MMS, Sao Jose BP, Sarker AR, Sarmiento-Suárez R, Sarrafzadegan N, Sartorius B, Sarvi S, Sathian B, Satpathy M, Sawant AR, Sawhney M, Saxena S, Sayyah M, Schaeffner E, Schmidt MI, Schneider IJC, Schöttker B, Schutte AE, Schwebel DC, Schwendicke F, Scott JG, Sekerija M, Sepanlou SG, Serván-Mori E, Seyedmousavi S, Shabaninejad H, Shackelford KA, Shafieesabet A, Shahbazi M, Shaheen AA, Shaikh MA, Shams-Beyranvand M, Shamsi M, Shamsizadeh M, Sharafi K, Sharif M, Sharif-Alhoseini M, Sharma R, She J, Sheikh A, Shi P, Shiferaw MS, Shigematsu M, Shiri R, Shirkoohi R, Shiue I, Shokraneh F, Shrime MG, Si S, Siabani S, Siddiqi TJ, Sigfusdottir ID, Sigurvinsdottir R, Silberberg DH, Silva DAS, Silva JP, Silva NTD, Silveira DGA, Singh JA, Singh NP, Singh PK, Singh V, Sinha DN, Sliwa K, Smith M, Sobaih BH, Sobhani S, Sobngwi E, Soneji SS, Soofi M, Sorensen RJD, Soriano JB, Soyiri IN, Sposato LA, Sreeramareddy CT, Srinivasan V, Stanaway JD, Starodubov VI, Stathopoulou V, Stein DJ, Steiner C, Stewart LG, Stokes MA, Subart ML, Sudaryanto A, Sufiyan MB, Sur PJ, Sutradhar I, Sykes BL, Sylaja PN, Sylte DO, Szoeke CEI, Tabarés-Seisdedos R, Tabuchi T, Tadakamadla SK, Takahashi K, Tandon N, Tassew SG, Taveira N, Tehrani-Banihashemi A, Tekalign TG, Tekle MG, Temsah M-H, Temsah O, Terkawi AS, Teshale MY, Tessema B, Tessema GA, Thankappan KR, Thirunavukkarasu S, Thomas N, Thrift AG, Thurston GD, Tilahun B, To QG, Tobe-Gai R, Tonelli M, Topor-Madry R, Torre AE, Tortajada-Girbés M, Touvier M, Tovani-Palone MR, Tran BX, Tran KB, Tripathi S, Troeger CE, Truelsen TC, Truong NT, Tsadik AG, Tsoi D, Tudor Car L, Tuzcu EM, Tyrovolas S, Ukwaja KN, Ullah I, Undurraga EA, Updike RL, Usman MS, Uthman OA, Uzun SB, Vaduganathan M, Vaezi A, Vaidya G, Valdez PR, Varavikova E, Vasankari TJ, Venketasubramanian N, Villafaina S, Violante FS, Vladimirov SK, Vlassov V, Vollset SE, Vos T, Wagner GR, Wagnew FS, Waheed Y, Wallin MT, Walson JL, Wang Y, Wang Y-P, Wassie MM, Weiderpass E, Weintraub RG, Weldegebreal F, Weldegwergs KG, Werdecker A, Werkneh AA, West TE, Westerman R, Whiteford HA, Widecka J, Wilner LB, Wilson S, Winkler AS, Wiysonge CS, Wolfe CDA, Wu S, Wu Y-C, Wyper GMA, Xavier D, Xu G, Yadgir S, Yadollahpour A, Yahyazadeh Jabbari SH, Yakob B, Yan LL, Yano Y, Yaseri M, Yasin YJ, Yentür GK, Yeshaneh A, Yimer EM, Yip P, Yirsaw BD, Yisma E, Yonemoto N, Yonga G, Yoon S-J, Yotebieng M, Younis MZ, Yousefifard M, Yu C, Zadnik V, Zaidi Z, Zaman SB, Zamani M, Zare Z, Zeleke AJ, Zenebe ZM, Zhang AL, Zhang K, Zhou M, Zodpey S, Zuhlke LJ, Naghavi M, Murray CJL. Global, regional, and national age-sex-specific mortality for 282 causes of death in 195 countries and territories, 1980-2017: a systematic analysis for the Global Burden of Disease Study 2017. Lancet 2018;392:1736–1788. 10.1016/s0140-6736(18)32203-7 30496103 PMC6227606

[2] Wilcox NS, Amit U, Reibel JB, Berlin E, Howell K, Ky B. Cardiovascular disease and cancer: shared risk factors and mechanisms. Nat Rev Cardiol 2024;21:617–631. 38600368 10.1038/s41569-024-01017-x PMC11324377

[3] Kort EJ, Paneth N, Woude GFV. The decline in US cancer mortality in people born since 1925. Cancer Res 2009;69:6500–6505. 19679548 10.1158/0008-5472.CAN-09-0357 PMC4326089

[4] Ng HS, Meng R, Marin TS, Damarell RA, Buckley E, Selvanayagam JB, Koczwara B. Cardiovascular mortality in people with cancer compared to the general population: a systematic review and meta-analysis. Cancer Med 2024;13:e70057. 39096123 10.1002/cam4.70057 PMC11297437

[5] Wang Z, Fan Z, Yang L, Liu L, Sheng C, Song F, Huang Y, Chen K. Higher risk of cardiovascular mortality than cancer mortality among long-term cancer survivors. Front Cardiovasc Med 2023;10:1014400. 36760569 10.3389/fcvm.2023.1014400 PMC9905625

[6] Kobo O, Raisi-Estabragh Z, Gevaert S, Rana JS, Van Spall HGC, Roguin A, Petersen SE, Ky B, Mamas MA. Impact of cancer diagnosis on distribution and trends of cardiovascular hospitalizations in the USA between 2004 and 2017. Eur Heart J Qual Care Clin Outcomes 2022;8:787–797. 35913736 10.1093/ehjqcco/qcac045 PMC9603542

[7] Matetic A, Mohamed M, Miller RJH, Kolman L, Lopez-Mattei J, Cheung WY, Brenner DR, Van Spall HGC, Graham M, Bianco C, Mamas MA. Impact of cancer diagnosis on causes and outcomes of 5.9 million US patients with cardiovascular admissions. Int J Cardiol 2021;341:76–83. 34333019 10.1016/j.ijcard.2021.07.054

[8] Agency for Healthcare Research and Quality (AHRQ) . HCUP-US NIS overview. https://www.hcup-us.ahrq.gov/ (January 2025).

[9] World Health Organization (WHO) . Ageing and health. https://www.who.int/news-room/fact-sheets/detail/ageing-and-health (January 2025).

[10] Salive ME . Multimorbidity in older adults. Epidemiol Rev 2013;35:75–83. 23372025 10.1093/epirev/mxs009

[11] Montégut L, López-Otín C, Kroemer G. Aging and cancer. Mol Cancer 2024;23:106. 38760832 10.1186/s12943-024-02020-z PMC11102267

[12] Lakatta EG, Levy D. Arterial and cardiac aging: major shareholders in cardiovascular disease enterprises: part I: aging arteries: a “set up” for vascular disease. Circulation 2003;107:139–146. 12515756 10.1161/01.cir.0000048892.83521.58

[13] Koo CY, Chong B, Jayabaskaran J, Nagarajan S, Jauhari SM, Chen Y, Tan LL, Mehta A, Khan MS, Muthiah M, Hausenloy D, Richards AM, Ko DT, Mallen CD, Secemsky EA, Chan MY, Thavendiranathan P, Chew NWS, Mamas MA. Global trends and forecast of cardiovascular diseases, cancer, and shared risk factors: insights from the GBD 2021. J Am Heart Assoc 2025;14:e043629. 41147370 10.1161/JAHA.125.043629 PMC12684631

[14] Okura Y, Takayama T, Ozaki K, Tanaka H, Kikuchi A, Saito T, Tanigawa T, Takii Y, Seki H, Takenouchi T, Chou T, Sato N, Tanabe N, Minamino T. Future projection of cancer patients with cardiovascular disease in Japan by the year 2039: a pilot study. Int J Clin Oncol 2019;24:983–994. 30903421 10.1007/s10147-019-01426-w PMC6597732

[15] Kachur S, Lavie CJ, de Schutter A, Milani RV, Ventura HO. Obesity and cardiovascular diseases. Minerva Med 2017;108:212–228. 28150485 10.23736/S0026-4806.17.05022-4

[16] World Obesity Federation . World Obesity Atlas 2025. London: World Obesity Federation; 2025. https://data.worldobesity.org/publications/world-obesity-atlas-2025-v6.pdf (April 2025).

[17] International Diabetes Federation (IDF) . IDF Diabetes Atlas. 11th ed. Brussels, Belgium: International Diabetes Federation (IDF); 2025. https://diabetesatlas.org/media/uploads/sites/3/2025/04/IDF_Atlas_11th_Edition_2025-1.pdf (April 2025).

[18] Centers for Disease Control and Prevention (CDC) . Lung cancer risk factors. https://www.cdc.gov/lung-cancer/risk-factors/index.html (March 2025).

[19] Münzel T, Hahad O, Kuntic M, Keaney JF, Deanfield JE, Daiber A. Effects of tobacco cigarettes, e-cigarettes, and waterpipe smoking on endothelial function and clinical outcomes. Eur Heart J 2020;41:4057–4070. 32585699 10.1093/eurheartj/ehaa460 PMC7454514

[20] Institute for Health Metrics and Evaluation (IHME) . Global Burden of Disease Study 2021 (GBD 2021). Smoking Mortality and Prevalence Forecasts 2022–2050. Seattle, United States of America: Institute for Health Metrics and Evaluation (IHME); 2024. https://ghdx.healthdata.org/record/ihme-data/gbd-2021-smoking-mortality-prevalence-forecasts-2022-2050 (April 2025).

[21] Coussens LM, Werb Z. Inflammation and cancer. Nature 2002;420:860–867. 12490959 10.1038/nature01322 PMC2803035

[22] Grivennikov SI, Greten FR, Karin M. Immunity, inflammation, and cancer. Cell 2010;140:883–899. 20303878 10.1016/j.cell.2010.01.025 PMC2866629

[23] Ross R . Atherosclerosis—an inflammatory disease. N Engl J Med 1999;340:115–126. 9887164 10.1056/NEJM199901143400207

[24] Perpinia AS, Kadoglou N, Vardaka M, Gkortzolidis G, Karavidas A, Marinakis T, Papachrysostomou C, Makaronis P, Vlachou C, Mantzourani M, Farmakis D, Konstantopoulos K. Pharmaceutical prevention and management of cardiotoxicity in hematological malignancies. Pharmaceuticals (Basel) 2022;15:1007. 36015155 10.3390/ph15081007 PMC9412591

[25] Tonorezos E, Devasia T, Mariotto AB, Mollica MA, Gallicchio L, Green P, Doose M, Brick R, Streck B, Reed C, de Moor JS. Prevalence of cancer survivors in the United States. J Natl Cancer Inst 2024;116:1784–1790. 39002121 10.1093/jnci/djae135 PMC11542986

[26] Florido R, Daya NR, Ndumele CE, Koton S, Russell SD, Prizment A, Blumenthal RS, Matsushita K, Mok Y, Felix AS, Coresh J, Joshu CE, Platz EA, Selvin E. Cardiovascular disease risk among cancer survivors. J Am Coll Cardiol 2022;80:22–32. 35772913 10.1016/j.jacc.2022.04.042 PMC9638987

[27] Tawfiq E, Pylypchuk R, Elwood JM, McKeage M, Wells S, Selak V. Risk of cardiovascular disease in cancer survivors: a cohort study of 446,384 New Zealand primary care patients. Cancer Med 2023;12:20081–20093. 37746882 10.1002/cam4.6580 PMC10587917

[28] Sung H, Ferlay J, Siegel RL, Laversanne M, Soerjomataram I, Jemal A, Bray F. Global cancer statistics 2020: GLOBOCAN estimates of incidence and mortality worldwide for 36 cancers in 185 countries. CA Cancer J Clin 2021;71:209–249. 33538338 10.3322/caac.21660

[29] International Agency for Research on Cancer (IARC)World Health Organization (WHO) . Global cancer burden growing, amidst mounting need for services. https://www.who.int/news/item/01-02-2024-global-cancer-burden-growing--amidst-mounting-need-for-services/ (January 2025).

[30] Weir HK, Thompson TD, Stewart SL, White MC. Cancer incidence projections in the United States between 2015 and 2050. Prev Chronic Dis 2021;18:E59. 34114543 10.5888/pcd18.210006 PMC8220959

[31] Rumgay H, Arnold M, Ferlay J, Lesi O, Cabasag CJ, Vignat J, Laversanne M, McGlynn KA, Soerjomataram I. Global burden of primary liver cancer in 2020 and predictions to 2040. J Hepatol 2022;77:1598–1606. 36208844 10.1016/j.jhep.2022.08.021 PMC9670241

[32] Burns SS, Kapur R. Putative mechanisms underlying cardiovascular disease associated with clonal hematopoiesis of indeterminate potential. Stem Cell Reports 2020;15:292–306. 32735822 10.1016/j.stemcr.2020.06.021 PMC7419714

[33] Zheng H, Mahmood SS, Khalique OK, Zhan H. Trastuzumab-induced cardiotoxicity: when and how much should we worry? JCO Oncol Pract 2024;20:1055–1063. 38662969 10.1200/OP.23.00816

[34] Ritter A, Quartermaine C, Pierre-Charles J, Balasubramanian S, Raeisi-Giglou P, Addison D, Miller E. Cardiotoxicity of anti-cancer radiation therapy: a focus on heart failure. Curr Heart Fail Rep 2023;20:44–55. 36692820 10.1007/s11897-023-00587-0

[35] Sørensen HT, Mellemkjaer L, Olsen JH, Baron JA. Prognosis of cancers associated with venous thromboembolism. N Engl J Med 2000;343:1846–1850. 11117976 10.1056/NEJM200012213432504

[36] Chitturi KR, Xu J, Araujo-Gutierrez R, Bhimaraj A, Guha A, Hussain I, Kassi M, Bernicker EH, Trachtenberg BH. Immune checkpoint inhibitor-related adverse cardiovascular events in patients with lung cancer. JACC CardioOncol 2019;1:182–192. 34396181 10.1016/j.jaccao.2019.11.013 PMC8352266

[37] Franquiz MJ, Waliany S, Xu AY, Hnatiuk A, Wu SM, Cheng P, Wakelee HA, Neal J, Witteles R, Zhu H. Osimertinib-associated cardiomyopathy in patients with non-small cell lung cancer: a case series. JACC CardioOncol 2023;5:839–841. 38205011 10.1016/j.jaccao.2023.07.006 PMC10774774

[38] Jacobs LA, Shulman LN. Cardio-oncology care delivery for all patients with cancer within academic and community settings. JACC CardioOncol 2024;6:470–472. 38983387 10.1016/j.jaccao.2023.10.010 PMC11229548

[39] Gilchrist SC, Barac A, Ades PA, Alfano CM, Franklin BA, Jones LW, La Gerche A, Ligibel JA, Lopez G, Madan K, Oeffinger KC, Salamone J, Scott JM, Squires RW, Thomas RJ, Treat-Jacobson DJ, Wright JS. Cardio-oncology rehabilitation to manage cardiovascular outcomes in cancer patients and survivors: a scientific statement from the American Heart Association. Circulation 2019;139:e997–e1012. 30955352 10.1161/CIR.0000000000000679 PMC7603804

[40] Lyon AR, López-Fernández T, Couch LS, Asteggiano R, Aznar MC, Bergler-Klein J, Boriani G, Cardinale D, Cordoba R, Cosyns B, Cutter DJ, de Azambuja E, de Boer RA, Dent SF, Farmakis D, Gevaert SA, Gorog DA, Herrmann J, Lenihan D, Moslehi J, Moura B, Salinger SS, Stephens R, Suter TM, Szmit S, Tamargo J, Thavendiranathan P, Tocchetti CG, van der Meer P, van der Pal HJH, Lancellotti P, Thuny F, Abdelhamid M, Aboyans V, Aleman B, Alexandre J, Barac A, Borger MA, Casado-Arroyo R, Cautela J, Čelutkienė J, Cikes M, Cohen-Solal A, Dhiman K, Ederhy S, Edvardsen T, Fauchier L, Fradley M, Grapsa J, Halvorsen S, Heuser M, Humbert M, Jaarsma T, Kahan T, Konradi A, Koskinas KC, Kotecha D, Ky B, Landmesser U, Lewis BS, Linhart A, Lip GYH, Løchen M-L, Malaczynska-Rajpold K, Metra M, Mindham R, Moonen M, Neilan TG, Nielsen JC, Petronio A-S, Prescott E, Rakisheva A, Salem J-E, Savarese G, Sitges M, Berg Jt, Touyz RM, Tycinska A, Wilhelm M, Zamorano JL, Laredj N, Zelveian P, Rainer PP, Samadov F, Andrushchuk U, Gerber BL, Selimović M, Kinova E, Samardzic J, Economides E, Pudil R, Nielsen KM, Kafafy TA, Vettus R, Tuohinen S, Ederhy S, Pagava Z, Rassaf T, Briasoulis A, Czuriga D, Andersen KK, Smyth Y, Iakobishvili Z, Parrini I, Rakisheva A, Pruthi EP, Mirrakhimov E, Kalejs O, Skouri H, Benlamin H, Žaliaduonytė D, Iovino A, Moore AM, Bursacovschi D, Benyass A, Manintveld O, Bosevski M, Gulati G, Leszek P, Fiuza M, Jurcut R, Vasyuk Y, Foscoli M, Simic D, Slanina M, Lipar L, Martin-Garcia A, Hübbert L, Kurmann R, Alayed A, Abid L, Zorkun C, Nesukay E, Manisty C, Srojidinova N, Baigent C, Abdelhamid M, Aboyans V, Antoniou S, Arbelo E, Asteggiano R, Baumbach A, Borger MA, Čelutkienė J, Cikes M, Collet J-P, Falk V, Fauchier L, Gale CP, Halvorsen S, Iung B, Jaarsma T, Konradi A, Koskinas KC, Kotecha D, Landmesser U, Lewis BS, Linhart A, Løchen M-L, Mindham R, Nielsen JC, Petersen SE, Prescott E, Rakisheva A, Sitges M, Touyz RM. 2022 ESC guidelines on cardio-oncology developed in collaboration with the European Hematology Association (EHA), the European Society for Therapeutic Radiology and Oncology (ESTRO) and the International Cardio-Oncology Society (IC-OS). Eur Heart J 2022;43:4229–4361. 36017568 10.1093/eurheartj/ehac244

[41] Adhikari BB, Shi S, Dimond EP, Shelburne N, Desvigne-Nickens P, Minasian LM. Spectrum of National Institutes of Health-Funded Research in Cardio-Oncology: a basic, clinical, and observational science perspective. Heart Fail Clin 2022;18:515–528. 35718423 10.1016/j.hfc.2022.01.001 PMC9328446

[42] Pareek N, Cevallos J, Moliner P, Shah M, Tan LL, Chambers V, Baksi AJ, Khattar RS, Sharma R, Rosen SD, Lyon AR. Activity and outcomes of a cardio-oncology service in the United Kingdom-a five-year experience. Eur J Heart Fail 2018;20:1721–1731. 30191649 10.1002/ejhf.1292

[43] Organisation for Economic Co-operation and Development (OECD) . Health at a Glance 2025: OECD Indicators. Paris: OECD Publishing; 2025.

[44] Busse R, Geissler A, Aaviksoo A, Cots F, Hakkinen U, Kobel C, Mateus C, Or Z, O'Reilly J, Serden L, Street A, Tan SS, Quentin W. Diagnosis related groups in Europe: moving towards transparency, efficiency, and quality in hospitals? BMJ 2013;346:f3197. 23747967 10.1136/bmj.f3197

