# Supplementary material for: Trends and future projections of cancer prevalence in patients with cardiovascular admissions
Source: Eur Heart J Open. 2026 Feb 19;6(2):oeag030. doi: 10.1093/ehjopen/oeag030 (PMC12989293; doi:10.1093/ehjopen/oeag030)
Supplement: oeag030_Supplementary_Data [file oeag030_supplementary_data.docx]

**SUPPLEMENTARY FILES**

**Supplementary Table 1.** Search codes.

| **Cancer types** | **Source** | **Codes** |
| --- | --- | --- |
| **All cancers** | ICD-10 | C00-C96 |
| **Breast cancer** | ICD-10 | C50 |
| **Colorectal cancer** | ICD-10 | C18-C21 |
| **Renal cancer** | ICD-10 | C64-C68 |
| **Liver cancer** | ICD-10 | C22 |
| **Haematological cancer** | ICD-10 | C81-C96 |
| **Lung and bronchus cancer** | ICD-10 | C34 |
| **Prostate and male genital cancer** | ICD-10 | C60-C63 |
| **Pancreatic cancer** | ICD-10 | C25 |
| **Female genital cancer** | ICD-10 | C51-C58 |
| **Skin cancer** | ICD-10 | C43-C44 |
| **Gastroesophageal cancer** | ICD-10 | C15-C16 |
| **Secondary unspecified** | ICD-10 | C76-C80 |
| **Other cancer** | ICD-10 | All other cancer codes |
| **Comorbidities** | **Source** | **Codes** |
| **Atrial fibrillation/flutter** | ICD-10 | I48.91, I48.20-21, I48.11, I48.19, I48.0 |
| **Dyslipidaemia** | ICD-10 | E78.x |
| **Smoking** | ICD-10 | Z72.0 |
| **Previous stroke** | ICD-10 | Z86.73 |
| **Previous AMI, PCI or CABG** | ICD-10 | I25.2; I25.6; Z98.61; Z95.5; Z95.1 |
| **Anaemias** | ICD-10 | D62.x, D63.x, D64.x |
| **Fluid and electrolyte disorders** | ICD-10 | E22.2; E86; E86.0; E86.1; E86.9; E87; E87.0; E87.1; E87.2; E87.3; E87.5; E87.6; E87.8; E87.7; E87.70; E87.71; E87.79 |
| **Hypertension** | ICD-10 | I10.x |
| **Peripheral artery disease** | ICD-10 | E08.5; E10.5; E11.5; E13.5; I70.2x; Z98.62 |
| **Diabetes mellitus** | ICD-10 | E08.x; E09.x; E10.x; E11.x; E13.x |
| **Coagulopathy** | ICD-10 | D65.x, D66.x, D67.x, D68.x, D69.x |
| **Depression** | ICD-10 | F32.x |
| **Dementia** | ICD-10 | F01; F01.5; F01.50; F01.51; F02; F02.8; F02.80;'F02.81; F03; F03.9; F03.90; F03.91; F05.1;'G30; G30.0; G30.1; G30.8; G30.9 |
| **Liver disease** | ICD-10 | K70.x, K72.1.x, K72.9.x, K73.x, K74.x, K75.x, K76.x, K77.x |
| **Chronic renal failure** | ICD-10 | N18.x |
| **Rheumatoid arthritis** | ICD-10 | M05.x-M06.x |
| **Valvular heart disease** | ICD-10 | I05; I06; I07; I08; I091; I34; I35; I36; I37; I38; I39 |
| **Alcohol or drug abuse** | ICD-10 | F10, G62.1, I42.6, K29.2, K70.0, K70.3, K70.9, Z50.2, Z71.4, Z72.1; F10, G62.1, I42.6, K29.2, K70.0, K70.3, K70.9, Z50.2, Z71.4, Z72.1 |
| **Obesity** | ICD-10 | E65, E66.x |
| **Hypothyroidism** | ICD-10 | E03.x |
| **Thrombocytopenia** | ICD-10 | D69.4.x, D69.5.x, D69.6.x |
| **Chronic pulmonary disease** | ICD-10 | J41.x, J42.x, J43.x, J44.x, J45.x, J47.x |
| **Chronic pancreatitis** | ICD-10 | K86.0, K86.1 |
| **AIDS** | ICD-10 | B20.x |
| **Metastatic disease** | ICD-10 | C77.x, C78.x, C79.x, R18.0.x, C7B.x |
| **Acute ischemic stroke** | ICD-10 | I63 |
| **Major bleeding** | ICD-10 | I60*, I61*, I62*, R58, K92.0, K92.1, K92.2 |

**Abbreviations:** ICD-10 – International Classification of Diseases Tenth Edition.

**Abbreviations:** None.

**Supplementary Table 2.** Absolute percentages of non-metastatic and metastatic cancers for each cancer type among all patients undergoing cardiovascular admissions across study period.

|  | Prevalence | Years | | | | | *P*-value* |
| --- | --- | --- | --- | --- | --- | --- | --- |
|  |  | 2016 | 2017 | 2018 | 2019 | 2020 |  |
| Overall cancer, % |  |  |  |  |  |  |  |
|  | Non-metastatic  Metastatic | 3.2  1.4 | 3.3  1.5 | 3.4  1.5 | 3.5  1.6 | 3.6  1.7 | 0.014 |
| Specific cancer type, % |  |  |  |  |  |  |  |
| Breast cancer | Non-metastatic  Metastatic | 0.2  0.1 | 0.2  0.1 | 0.3  0.1 | 0.3  0.1 | 0.3  0.1 | 0.686 |
| Colorectal cancer | Non-metastatic  Metastatic | 0.1  0.1 | 0.1  0.1 | 0.2  0.1 | 0.2  0.1 | 0.2  0.1 | 0.163 |
| Renal cancer | Non-metastatic  Metastatic | 0.2  0.1 | 0.2  0.1 | 0.2  0.1 | 0.3  0.1 | 0.3  0.1 | 0.828 |
| Liver cancer | Non-metastatic  Metastatic | 0.1  <0.1 | 0.1  <0.1 | 0.1  <0.1 | 0.1  <0.1 | 0.1  <0.1 | 0.097 |
| Haematological cancer | Non-metastatic  Metastatic | 1.2  <0.1 | 1.2  <0.1 | 1.2  <0.1 | 1.3  <0.1 | 1.3  <0.1 | 0.034 |
| Lung and bronchus cancer | Non-metastatic  Metastatic | 0.5  0.3 | 0.5  0.3 | 0.5  0.3 | 0.5  0.3 | 0.5  0.4 | 0.023 |
| Prostate and male genital cancer | Non-metastatic  Metastatic | 0.3  0.1 | 0.3  0.1 | 0.3  0.1 | 0.4  0.2 | 0.4  0.2 | 0.108 |
| Pancreatic cancer | Non-metastatic  Metastatic | 0.1  0.1 | 0.1  0.1 | 0.1  0.1 | 0.1  0.1 | 0.1  0.1 | 0.094 |
| Female genital cancer | Non-metastatic  Metastatic | 0.1  0.1 | 0.1  0.1 | 0.1  0.1 | 0.1  0.1 | 0.1  0.1 | 0.023 |
| Skin cancer | Non-metastatic  Metastatic | 0.1  <0.1 | 0.1  <0.1 | 0.1  <0.1 | 0.1  <0.1 | 0.1  <0.1 | 0.001 |
| Gastroesophageal cancer | Non-metastatic  Metastatic | 0.1  <0.1 | 0.1  <0.1 | 0.1  <0.1 | 0.1  <0.1 | 0.1  <0.1 | 0.443 |
| Secondary unspecified cancer | Non-metastatic  Metastatic | <0.1  0.4 | <0.1  0.5 | <0.1  0.5 | <0.1  0.5 | <0.1  0.5 | 0.630 |
| Other cancer | Non-metastatic  Metastatic | 0.2  <0.1 | 0.2  <0.1 | 0.2  <0.1 | 0.2  <0.1 | 0.2  <0.1 | 0.581 |

*Mantel-Haenszel test for trend

All analyses were weighted using the provided discharge weights as recommended by HCUP. The variables “HOSP_NIS” and “NIS_Stratum” were used for clustering and stratification of the data, respectively.

**Abbreviations:** None.

**Supplementary Table 3.** Relative percentages of non-metastatic and metastatic cancers for each cancer type among cancer patients undergoing cardiovascular admissions across study period.

|  | Prevalence | Years | | | | | *P*-value* |
| --- | --- | --- | --- | --- | --- | --- | --- |
|  |  | 2016 | 2017 | 2018 | 2019 | 2020 |  |
| Overall cancer, % |  |  |  |  |  |  |  |
|  | Non-metastatic  Metastatic | 68.9  31.1 | 68.3  31.7 | 68.7  31.3 | 68.6  31.4 | 67.9  32.1 | 0.014 |
| Specific cancer type, % |  |  |  |  |  |  |  |
| Breast cancer | Non-metastatic  Metastatic | 66.8  33.2 | 66.3  33.7 | 67.6  32.4 | 66.1  33.9 | 67.5  32.5 | 0.686 |
| Colorectal cancer | Non-metastatic  Metastatic | 57.4  42.6 | 56.7  43.3 | 59.5  40.5 | 57.5  42.5 | 58.1  41.9 | 0.163 |
| Renal cancer | Non-metastatic  Metastatic | 74.1  25.9 | 75.5  24.5 | 74.4  25.6 | 75.7  24.3 | 73.6  26.4 | 0.828 |
| Liver cancer | Non-metastatic  Metastatic | 78.8  21.2 | 76.5  23.5 | 77.0  23.0 | 75.0  25.0 | 76.3  23.7 | 0.097 |
| Haematological cancer | Non-metastatic  Metastatic | 97.5  2.5 | 97.9  2.1 | 97.7  2.3 | 97.5  2.5 | 97.3  2.7 | 0.034 |
| Lung and bronchus cancer | Non-metastatic  Metastatic | 60.9  39.1 | 60.2  39.8 | 60.5  39.5 | 60.5  39.5 | 58.6  41.4 | 0.023 |
| Prostate and male genital cancer | Non-metastatic  Metastatic | 70.8  29.2 | 68.5  31.5 | 69.5  30.5 | 69.3  30.7 | 68.6  31.4 | 0.108 |
| Pancreatic cancer | Non-metastatic  Metastatic | 41.3  58.7 | 40.8  59.2 | 41.1  58.9 | 38.6  61.4 | 39.1  60.9 | 0.094 |
| Female genital cancer | Non-metastatic  Metastatic | 62.2  37.8 | 58.3  41.7 | 61.1  38.9 | 59.2  40.8 | 57.5  42.5 | 0.023 |
| Skin cancer | Non-metastatic  Metastatic | 79.2  20.8 | 79.5  20.5 | 77.4  22.6 | 77.8  22.2 | 73.5  26.5 | 0.001 |
| Gastroesophageal cancer | Non-metastatic  Metastatic | 62.6  37.4 | 64.9  35.1 | 63.8  36.2 | 61.7  38.3 | 62.4  37.6 | 0.443 |
| Secondary unspecified cancer | Non-metastatic  Metastatic | 6.6  93.4 | 5.6  94.4 | 6.0  94.0 | 6.3  93.7 | 5.9  94.1 | 0.630 |
| Other cancer | Non-metastatic  Metastatic | 99.6  0.4 | 99.8  0.2 | 99.9  0.1 | 99.8  0.2 | 99.7  0.3 | 0.581 |

*Mantel-Haenszel test for trend

All analyses were weighted using the provided discharge weights as recommended by HCUP. The variables “HOSP_NIS” and “NIS_Stratum” were used for clustering and stratification of the data, respectively.

**Abbreviations:** None.

**Supplementary Table 4.** **Prevalence of specific cardiovascular admissions** in patients undergoing cardiovascular admissions across study period.

| Prevalence | Years | | | | | *P*-value* |
| --- | --- | --- | --- | --- | --- | --- |
|  | **2016** | **2017** | **2018** | **2019** | **2020** |  |
| Specific cardiovascular admission, % |  |  |  |  |  |  |
| ACS | 17.2 | 16.8 | 15.8 | 15.5 | 15.8 | <0.001 |
| Heart failure | 22.8 | 24.5 | 25.5 | 26.0 | 26.0 | <0.001 |
| Hypertension and complications (excluding heart failure) | 4.5 | 4.6 | 4.6 | 4.7 | 4.8 | <0.001 |
| Atrial fibrillation/flutter | 9.7 | 9.5 | 9.5 | 9.1 | 7.0 | <0.001 |
| Other arrhythmias | 4.3 | 4.3 | 4.3 | 4.3 | 4.4 | <0.001 |
| Pulmonary embolism | 3.9 | 3.8 | 3.8 | 3.7 | 4.2 | <0.001 |
| Ischemic stroke | 10.6 | 10.7 | 10.7 | 11.0 | 11.9 | <0.001 |
| Haemorrhagic stroke | 2.7 | 2.7 | 2.6 | 2.6 | 2.9 | <0.001 |
| Valve disorders | 2.7 | 2.8 | 2.9 | 3.1 | 3.3 | <0.001 |
| Other cardiovascular causes | 21.6 | 20.4 | 20.3 | 20.0 | 19.7 |  |

*Mantel-Haenszel test for trend

All analyses were weighted using the provided discharge weights as recommended by HCUP. The variables “HOSP_NIS” and “NIS_Stratum” were used for clustering and stratification of the data, respectively.

**Abbreviations:** None.

**Supplementary Table 5.** **Prediction of** **cancer prevalence** in patients undergoing cardiovascular admissions until 2040.

| Prevalence | Years | | | | |  |  |  |  |  |  |  |  |  |  |  |  |  |  |  |  |  |  |  |  | *P*-value |
| --- | --- | --- | --- | --- | --- | --- | --- | --- | --- | --- | --- | --- | --- | --- | --- | --- | --- | --- | --- | --- | --- | --- | --- | --- | --- | --- |
|  | **2016** | **2017** | **2018** | **2019** | **2020** | **2021** | **2022** | **2023** | **2024** | **2025** | **2026** | **2027** | **2028** | **2029** | **2030** | **2031** | **2032** | **2033** | **2034** | **2035** | **2036** | **2037** | **2038** | **2039** | **2040** |  |
| Overall cancer, % | 4.8 | 5.0 | 5.1 | 5.3 | 5.4 | 5.7 | 6.0 | 6.2 | 6.5 | 6.6 | 6.8 | 7.1 | 7.5 | 7.8 | 8.0 | 8.4 | 8.4 | 9.1 | 9.5 | 9.8 | 10.1 | 10.5 | 11.1 | 11.3 | 11.9 |  |
| Specific cancer type, % |  |  |  |  |  |  |  |  |  |  |  |  |  |  |  |  |  |  |  |  |  |  |  |  |  |  |
| Breast cancer | 0.3 | 0.4 | 0.4 | 0.4 | 0.4 | 0.5 | 0.5 | 0.5 | 0.5 | 0.6 | 0.6 | 0.6 | 0.7 | 0.7 | 0.7 | 0.8 | 0.8 | 0.9 | 0.9 | 1.0 | 1.0 | 1.1 | 1.2 | 1.2 | 1.3 | <0.001 |
| Colorectal cancer | 0.3 | 0.3 | 0.3 | 0.3 | 0.3 | 0.3 | 0.3 | 0.4 | 0.4 | 0.4 | 0.4 | 0.4 | 0.4 | 0.5 | 0.5 | 0.5 | 0.5 | 0.5 | 0.6 | 0.6 | 0.6 | 0.6 | 0.7 | 0.7 | 0.7 | <0.001 |
| Renal cancer | 0.3 | 0.3 | 0.3 | 0.4 | 0.4 | 0.4 | 0.4 | 0.4 | 0.5 | 0.5 | 0.5 | 0.5 | 0.6 | 0.6 | 0.6 | 0.7 | 0.7 | 0.8 | 0.8 | 0.8 | 0.9 | 0.9 | 1.0 | 1.0 | 1.1 | <0.001 |
| Liver cancer | 0.1 | 0.1 | 0.1 | 0.1 | 0.1 | 0.2 | 0.2 | 0.2 | 0.2 | 0.2 | 0.2 | 0.2 | 0.3 | 0.3 | 0.3 | 0.3 | 0.3 | 0.3 | 0.4 | 0.4 | 0.4 | 0.5 | 0.5 | 0.5 | 0.6 | <0.001 |
| Haematological cancer | 1.2 | 1.3 | 1.3 | 1.4 | 1.4 | 1.4 | 1.5 | 1.5 | 1.6 | 1.6 | 1.7 | 1.7 | 1.8 | 1.8 | 1.9 | 2.0 | 2.0 | 2.1 | 2.2 | 2.2 | 2.3 | 2.4 | 2.4 | 2.5 | 2.6 | <0.001 |
| Lung and bronchus cancer | 0.8 | 0.8 | 0.9 | 0.9 | 0.9 | 0.9 | 0.9 | 1.0 | 1.0 | 1.0 | 1.1 | 1.1 | 1.1 | 1.2 | 1.2 | 1.2 | 1.2 | 1.3 | 1.3 | 1.4 | 1.4 | 1.4 | 1.5 | 1.5 | 1.6 | <0.001 |
| Prostate and male genital cancer | 0.5 | 0.5 | 0.5 | 0.5 | 0.6 | 0.6 | 0.6 | 0.6 | 0.7 | 0.7 | 0.7 | 0.8 | 0.8 | 0.8 | 0.9 | 0.9 | 0.9 | 1.0 | 1.0 | 1.1 | 1.1 | 1.1 | 1.2 | 1.2 | 1.3 | <0.001 |
| Pancreatic cancer | 0.2 | 0.2 | 0.2 | 0.2 | 0.2 | 0.2 | 0.2 | 0.2 | 0.2 | 0.2 | 0.2 | 0.3 | 0.3 | 0.3 | 0.3 | 0.3 | 0.3 | 0.3 | 0.4 | 0.4 | 0.4 | 0.4 | 0.4 | 0.5 | 0.5 | <0.001 |
| Female genital cancer | 0.2 | 0.2 | 0.2 | 0.2 | 0.2 | 0.2 | 0.2 | 0.2 | 0.2 | 0.2 | 0.2 | 0.2 | 0.2 | 0.2 | 0.2 | 0.2 | 0.2 | 0.3 | 0.3 | 0.3 | 0.3 | 0.3 | 0.3 | 0.3 | 0.3 | 0.031 |
| Skin cancer | 0.1 | 0.1 | 0.1 | 0.1 | 0.1 | 0.1 | 0.2 | 0.2 | 0.2 | 0.2 | 0.2 | 0.2 | 0.2 | 0.2 | 0.2 | 0.2 | 0.2 | 0.2 | 0.2 | 0.2 | 0.3 | 0.3 | 0.3 | 0.3 | 0.3 | <0.001 |
| Gastroesophageal cancer | 0.1 | 0.1 | 0.1 | 0.1 | 0.1 | 0.1 | 0.1 | 0.1 | 0.1 | 0.1 | 0.1 | 0.1 | 0.1 | 0.2 | 0.2 | 0.2 | 0.2 | 0.2 | 0.2 | 0.2 | 0.2 | 0.2 | 0.2 | 0.2 | 0.2 | <0.001 |
| Secondary unspecified | 0.5 | 0.5 | 0.5 | 0.5 | 0.5 | 0.6 | 0.6 | 0.6 | 0.6 | 0.6 | 0.6 | 0.7 | 0.7 | 0.7 | 0.7 | 0.7 | 0.7 | 0.8 | 0.8 | 0.8 | 0.8 | 0.8 | 0.9 | 0.9 | 0.9 | <0.001 |
| Other cancer | 0.2 | 0.2 | 0.2 | 0.2 | 0.2 | 0.2 | 0.3 | 0.3 | 0.3 | 0.3 | 0.3 | 0.3 | 0.3 | 0.3 | 0.3 | 0.4 | 0.4 | 0.4 | 0.4 | 0.4 | 0.4 | 0.5 | 0.5 | 0.5 | 0.5 | <0.001 |

All analyses were weighted using the provided discharge weights as recommended by HCUP. The variables “HOSP_NIS” and “NIS_Stratum” were used for clustering and stratification of the data, respectively.

**Abbreviations:** None.

**Supplementary Table 6A. Prediction of** **cancer prevalence** in patients undergoing cardiovascular admissions until 2040, including Confidence Intervals and Standard Error.

| Years | Breast Cancer  (IRR 1.056, 95% CI 1.045 – 1.068, P<0.001) | | | Colorectal Cancer  (IRR 1.044, 95% CI 1.031 – 1.056, P<0.001) | | | Renal Cancer  (IRR 1.055, 95% CI 1.043 – 1.067, P<0.001) | | | Liver Cancer  (IRR 1.069, 95% CI 1.049 – 1.089, P<0.001) | | | | |
| --- | --- | --- | --- | --- | --- | --- | --- | --- | --- | --- | --- | --- | --- | --- |
|  | **Rate** | **Standard Error** | **[95% CI]** | **Rate** | **Standard Error** | **[95% CI]** | **Rate** | **Standard Error** | **[95% CI]** | **Rate** | | **Standard Error** | | **[95% CI]** |
| 2016 | 0.3 | 0.005 | 0.337, 0.356 | 0.3 | 0.004 | 0.257, 0.273 | 0.3 | 0.004 | 0.293, 0.310 | 0.1 | 0.003 | | 0.107, 0.118 | |
| 2017 | 0.4 | 0.003 | 0.359, 0.373 | 0.3 | 0.003 | 0.271, 0.283 | 0.3 | 0.003 | 0.312, 0.325 | 0.1 | 0.002 | | 0.116, 0.124 | |
| 2018 | 0.4 | 0.003 | 0.381, 0.392 | 0.3 | 0.003 | 0.284, 0.294 | 0.3 | 0.003 | 0.331, 0.341 | 0.1 | 0.002 | | 0.125, 0.132 | |
| 2019 | 0.4 | 0.004 | 0.401, 0.416 | 0.3 | 0.003 | 0.295, 0.307 | 0.4 | 0.003 | 0.348, 0.361 | 0.1 | 0.002 | | 0.133, 0.142 | |
| 2020 | 0.4 | 0.006 | 0.420, 0.442 | 0.3 | 0.005 | 0.305, 0.324 | 0.4 | 0.005 | 0.364, 0.384 | 0.1 | 0.003 | | 0.141, 0.153 | |
| 2021 | 0.5 | 0.008 | 0.439, 0.471 | 0.3 | 0.007 | 0.315, 0.341 | 0.4 | 0.007 | 0.380, 0.409 | 0.2 | 0.005 | | 0.148, 0.166 | |
| 2022 | 0.5 | 0.011 | 0.460, 0.503 | 0.3 | 0.009 | 0.325, 0.360 | 0.4 | 0.010 | 0.397, 0.436 | 0.2 | 0.007 | | 0.155, 0.181 | |
| 2023 | 0.5 | 0.014 | 0.481, 0.536 | 0.4 | 0.012 | 0.335, 0.380 | 0.4 | 0.013 | 0.414, 0.465 | 0.2 | 0.009 | | 0.162, 0.196 | |
| 2024 | 0.5 | 0.018 | 0.502, 0.572 | 0.4 | 0.014 | 0.345, 0.401 | 0.5 | 0.016 | 0.432, 0.496 | 0.2 | 0.011 | | 0.170, 0.213 | |
| 2025 | 0.6 | 0.022 | 0.525, 0.609 | 0.4 | 0.017 | 0.355, 0.423 | 0.5 | 0.020 | 0.450, 0.529 | 0.2 | 0.014 | | 0.178, 0.231 | |
| 2026 | 0.6 | 0.026 | 0.548, 0.650 | 0.4 | 0.021 | 0.366, 0.446 | 0.5 | 0.024 | 0.469, 0.564 | 0.2 | 0.017 | | 0.186, 0.251 | |
| 2027 | 0.6 | 0.031 | 0.572, 0.694 | 0.4 | 0.024 | 0.376, 0.471 | 0.5 | 0.029 | 0.489, 0.601 | 0.2 | 0.020 | | 0.195, 0.273 | |
| 2028 | 0.7 | 0.036 | 0.597, 0.740 | 0.4 | 0.028 | 0.388, 0.497 | 0.6 | 0.034 | 0.509, 0.641 | 0.3 | 0.024 | | 0.204, 0.296 | |
| 2029 | 0.7 | 0.042 | 0.623, 0.789 | 0.5 | 0.032 | 0.399, 0.524 | 0.6 | 0.039 | 0.531, 0.683 | 0.3 | 0.028 | | 0.213, 0.321 | |
| 2030 | 0.7 | 0.049 | 0.651, 0.841 | 0.5 | 0.036 | 0.410, 0.553 | 0.6 | 0.045 | 0.553, 0.728 | 0.3 | 0.032 | | 0.222, 0.349 | |
| 2031 | 0.8 | 0.056 | 0.679, 0.896 | 0.5 | 0.041 | 0.422, 0.583 | 0.7 | 0.051 | 0.576, 0.776 | 0.3 | 0.037 | | 0.232, 0.378 | |
| 2032 | 0.8 | 0.063 | 0.708, 0.956 | 0.5 | 0.046 | 0.434, 0.615 | 0.7 | 0.058 | 0.599, 0.827 | 0.3 | 0.043 | | 0.242, 0.410 | |
| 2033 | 0.9 | 0.071 | 0.739, 1.019 | 0.5 | 0.052 | 0.446, 0.648 | 0.8 | 0.066 | 0.624, 0.881 | 0.3 | 0.049 | | 0.252, 0.445 | |
| 2034 | 0.9 | 0.080 | 0.771, 1.086 | 0.6 | 0.057 | 0.459, 0.683 | 0.8 | 0.074 | 0.649, 0.939 | 0.4 | 0.056 | | 0.263, 0.482 | |
| 2035 | 1.0 | 0.090 | 0.804, 1.157 | 0.6 | 0.064 | 0.471, 0.721 | 0.8 | 0.083 | 0.676, 1.000 | 0.4 | 0.063 | | 0.274, 0.523 | |
| 2036 | 1.0 | 0.101 | 0.838, 1.233 | 0.6 | 0.070 | 0.484, 0.760 | 0.9 | 0.092 | 0.703, 1.065 | 0.4 | 0.072 | | 0.285, 0.566 | |
| 2037 | 1.1 | 0.113 | 0.873, 1.314 | 0.6 | 0.077 | 0.497, 0.801 | 0.9 | 0.103 | 0.731, 1.135 | 0.5 | 0.081 | | 0.296, 0.614 | |
| 2038 | 1.2 | 0.125 | 0.910, 1.401 | 0.7 | 0.085 | 0.511, 0.844 | 1.0 | 0.114 | 0.760, 1.209 | 0.5 | 0.091 | | 0.307, 0.665 | |
| 2039 | 1.2 | 0.139 | 0.949, 1.492 | 0.7 | 0.093 | 0.524, 0.889 | 1.0 | 0.127 | 0.790, 1.287 | 0.5 | 0.102 | | 0.319, 0.720 | |
| 2040 | 1.3 | 0.154 | 0.988, 1.590 | 0.7 | 0.102 | 0.538, 0.937 | 1.1 | 0.140 | 0.822, 1.371 | 0.6 | 0.115 | | 0.330, 0.780 | |

All analyses were weighted using the provided discharge weights as recommended by HCUP. The variables “HOSP_NIS” and “NIS_Stratum” were used for clustering and stratification of the data, respectively.

Rates represent model-based fitted values for 2016-2020 and projected values for 2021-2040 derived from Poisson regression.

Standard Errors and Confidence Intervals are reported with sufficient decimal precision to avoid loss of information.

**Abbreviations:** None.

**Supplementary Table 6B.** **Prediction of** **cancer prevalence** in patients undergoing cardiovascular admissions until 2040, including Confidence Intervals and Standard Error.

| Years | Haematological Cancer  (IRR 1.032, 95% CI 1.026 – 1.037, P<0.001) | | | Lung Cancer  (IRR 1.028, 95% CI 1.021 – 1.035, P<0.001) | | | Prostate Cancer  (IRR 1.042, 95% CI 1.033 – 1.052, P<0.001) | | | Pancreatic Cancer  (IRR 1.049, 95% CI 1.032 – 1.066, P<0.001) | | |
| --- | --- | --- | --- | --- | --- | --- | --- | --- | --- | --- | --- | --- |
|  | **Rate** | **Standard Error** | **[95% CI]** | **Rate** | **Standard Error** | **[95% CI]** | **Rate** | **Standard Error** | **[95% CI]** | **Rate** | **Standard Error** | **[95% CI]** |
| 2016 | 1.2 | 0.009 | 1.214, 1.249 | 0.8 | 0.007 | 0.791, 0.819 | 0.5 | 0.006 | 0.468, 0.489 | 0.2 | 0.003 | 0.147, 0.159 |
| 2017 | 1.3 | 0.006 | 1.258, 1.283 | 0.8 | 0.005 | 0.817, 0.837 | 0.5 | 0.004 | 0.491, 0.507 | 0.2 | 0.002 | 0.156, 0.165 |
| 2018 | 1.3 | 0.005 | 1.300, 1.321 | 0.9 | 0.004 | 0.842, 0.859 | 0.5 | 0.003 | 0.513, 0.526 | 0.2 | 0.002 | 0.165, 0.172 |
| 2019 | 1.4 | 0.007 | 1.339, 1.365 | 0.9 | 0.005 | 0.863, 0.885 | 0.5 | 0.004 | 0.533, 0.550 | 0.2 | 0.002 | 0.172, 0.182 |
| 2020 | 1.4 | 0.010 | 1.375, 1.414 | 0.9 | 0.008 | 0.883, 0.914 | 0.6 | 0.006 | 0.552, 0.577 | 0.2 | 0.004 | 0.179, 0.193 |
| 2021 | 1.4 | 0.014 | 1.411, 1.466 | 0.9 | 0.011 | 0.902, 0.945 | 0.6 | 0.009 | 0.571, 0.606 | 0.2 | 0.005 | 0.185, 0.205 |
| 2022 | 1.5 | 0.018 | 1.448, 1.520 | 0.9 | 0.015 | 0.921, 0.978 | 0.6 | 0.012 | 0.590, 0.637 | 0.2 | 0.007 | 0.191, 0.218 |
| 2023 | 1.5 | 0.023 | 1.486, 1.576 | 1.0 | 0.018 | 0.940, 1.012 | 0.6 | 0.015 | 0.609, 0.670 | 0.2 | 0.009 | 0.197, 0.232 |
| 2024 | 1.6 | 0.028 | 1.523, 1.635 | 1.0 | 0.022 | 0.959, 1.047 | 0.7 | 0.019 | 0.629, 0.704 | 0.2 | 0.011 | 0.203, 0.247 |
| 2025 | 1.6 | 0.034 | 1.562, 1.695 | 1.0 | 0.027 | 0.979, 1.083 | 0.7 | 0.023 | 0.650, 0.740 | 0.2 | 0.014 | 0.209, 0.263 |
| 2026 | 1.7 | 0.040 | 1.602, 1.758 | 1.1 | 0.031 | 0.998, 1.121 | 0.7 | 0.027 | 0.671, 0.778 | 0.2 | 0.016 | 0.216, 0.280 |
| 2027 | 1.7 | 0.046 | 1.643, 1.823 | 1.1 | 0.036 | 1.019, 1.160 | 0.8 | 0.032 | 0.692, 0.818 | 0.3 | 0.019 | 0.222, 0.298 |
| 2028 | 1.8 | 0.053 | 1.684, 1.891 | 1.1 | 0.041 | 1.039, 1.200 | 0.8 | 0.037 | 0.715, 0.859 | 0.3 | 0.022 | 0.229, 0.317 |
| 2029 | 1.8 | 0.060 | 1.727, 1.961 | 1.2 | 0.046 | 1.060, 1.242 | 0.8 | 0.042 | 0.737, 0.903 | 0.3 | 0.026 | 0.235, 0.337 |
| 2030 | 1.9 | 0.067 | 1.770, 2.034 | 1.2 | 0.052 | 1.081, 1.285 | 0.9 | 0.048 | 0.761, 0.949 | 0.3 | 0.029 | 0.242, 0.358 |
| 2031 | 2.0 | 0.075 | 1.815, 2.109 | 1.2 | 0.058 | 1.103, 1.329 | 0.9 | 0.054 | 0.785, 0.998 | 0.3 | 0.034 | 0.249, 0.381 |
| 2032 | 2.0 | 0.083 | 1.861, 2.187 | 1.2 | 0.064 | 1.125, 1.375 | 0.9 | 0.061 | 0.810, 1.048 | 0.3 | 0.038 | 0.256, 0.405 |
| 2033 | 2.1 | 0.092 | 1.907, 2.268 | 1.3 | 0.070 | 1.147, 1.423 | 1.0 | 0.068 | 0.835, 1.101 | 0.3 | 0.043 | 0.263, 0.431 |
| 2034 | 2.2 | 0.101 | 1.955, 2.352 | 1.3 | 0.077 | 1.169, 1.472 | 1.0 | 0.075 | 0.861, 1.157 | 0.4 | 0.048 | 0.270, 0.458 |
| 2035 | 2.2 | 0.111 | 2.004, 2.439 | 1.4 | 0.084 | 1.192, 1.523 | 1.1 | 0.084 | 0.888, 1.216 | 0.4 | 0.053 | 0.277, 0.486 |
| 2036 | 2.3 | 0.121 | 2.054, 2.528 | 1.4 | 0.092 | 1.216, 1.575 | 1.1 | 0.092 | 0.916, 1.277 | 0.4 | 0.059 | 0.284, 0.517 |
| 2037 | 2.4 | 0.132 | 2.106, 2.622 | 1.4 | 0.099 | 1.239, 1.629 | 1.1 | 0.101 | 0.944, 1.342 | 0.4 | 0.066 | 0.292, 0.549 |
| 2038 | 2.4 | 0.143 | 2.158, 2.718 | 1.5 | 0.108 | 1.264, 1.685 | 1.2 | 0.111 | 0.973, 1.410 | 0.4 | 0.072 | 0.299, 0.583 |
| 2039 | 2.5 | 0.155 | 2.212, 2.818 | 1.5 | 0.116 | 1.288, 1.743 | 1.2 | 0.122 | 1.003, 1.481 | 0.5 | 0.080 | 0.306, 0.619 |
| 2040 | 2.6 | 0.167 | 2.266, 2.922 | 1.6 | 0.125 | 1.313, 1.803 | 1.3 | 0.133 | 1.034, 1.555 | 0.5 | 0.088 | 0.314, 0.657 |

All analyses were weighted using the provided discharge weights as recommended by HCUP. The variables “HOSP_NIS” and “NIS_Stratum” were used for clustering and stratification of the data, respectively.

Rates represent model-based fitted values for 2016-2020 and projected values for 2021-2040 derived from Poisson regression.

Standard Errors and Confidence Intervals are reported with sufficient decimal precision to avoid loss of information.

**Abbreviations:** None.

**Supplementary Table 6C.** **Prediction of** **cancer prevalence** in patients undergoing cardiovascular admissions until 2040, including Confidence Intervals and Standard Error.

| Years | Female genital Cancer  (IRR 1.017, 95% CI 1.015 – 1.032, P=0.031) | | | Skin Cancer  (IRR 1.038, 95% CI 1.019 – 1.057, P<0.001) | | | Gastroesophageal Cancer  (IRR 1.038, 95% CI 1.017 – 1.060, P<0.001) | | | Secondary Unspecified Cancer  (IRR 1.026, 95% CI 1.017 – 1.036, P<0.001) | | |
| --- | --- | --- | --- | --- | --- | --- | --- | --- | --- | --- | --- | --- |
|  | **Rate** | **Standard Error** | **[95% CI]** | **Rate** | **Standard Error** | **[95% CI]** | **Rate** | **Standard Error** | **[95% CI]** | **Rate** | **Standard Error** | **[95% CI]** |
| 2016 | 0.2 | 0.004 | 0.183, 0.197 | 0.1 | 0.003 | 0.114, 0.125 | 0.1 | 0.002 | 0.088, 0.097 | 0.5 | 0.006 | 0.480, 0.502 |
| 2017 | 0.2 | 0.003 | 0.188, 0.198 | 0.1 | 0.002 | 0.120, 0.128 | 0.1 | 0.002 | 0.093, 0.100 | 0.5 | 0.004 | 0.496, 0.511 |
| 2018 | 0.2 | 0.002 | 0.192, 0.201 | 0.1 | 0.002 | 0.125, 0.132 | 0.1 | 0.001 | 0.097, 0.103 | 0.5 | 0.003 | 0.510, 0.523 |
| 2019 | 0.2 | 0.003 | 0.195, 0.205 | 0.1 | 0.002 | 0.129, 0.138 | 0.1 | 0.002 | 0.100, 0.107 | 0.5 | 0.004 | 0.522, 0.539 |
| 2020 | 0.2 | 0.004 | 0.196, 0.210 | 0.1 | 0.003 | 0.132, 0.145 | 0.1 | 0.003 | 0.102, 0.113 | 0.5 | 0.006 | 0.532, 0.557 |
| 2021 | 0.2 | 0.005 | 0.196, 0.217 | 0.1 | 0.004 | 0.135, 0.152 | 0.1 | 0.004 | 0.104, 0.119 | 0.6 | 0.009 | 0.542, 0.576 |
| 2022 | 0.2 | 0.007 | 0.197, 0.223 | 0.2 | 0.006 | 0.138, 0.161 | 0.1 | 0.005 | 0.106, 0.126 | 0.6 | 0.011 | 0.551, 0.596 |
| 2023 | 0.2 | 0.008 | 0.197, 0.230 | 0.2 | 0.008 | 0.140, 0.170 | 0.1 | 0.007 | 0.107, 0.134 | 0.6 | 0.014 | 0.561, 0.617 |
| 2024 | 0.2 | 0.010 | 0.197, 0.237 | 0.2 | 0.009 | 0.143, 0.179 | 0.1 | 0.008 | 0.109, 0.141 | 0.6 | 0.017 | 0.570, 0.639 |
| 2025 | 0.2 | 0.012 | 0.197, 0.244 | 0.2 | 0.011 | 0.145, 0.189 | 0.1 | 0.010 | 0.111, 0.149 | 0.6 | 0.021 | 0.580, 0.661 |
| 2026 | 0.2 | 0.014 | 0.197, 0.251 | 0.2 | 0.013 | 0.147, 0.199 | 0.1 | 0.012 | 0.112, 0.158 | 0.6 | 0.024 | 0.589, 0.684 |
| 2027 | 0.2 | 0.016 | 0.197, 0.259 | 0.2 | 0.015 | 0.150, 0.210 | 0.1 | 0.014 | 0.114, 0.167 | 0.7 | 0.028 | 0.599, 0.708 |
| 2028 | 0.2 | 0.018 | 0.197, 0.266 | 0.2 | 0.018 | 0.152, 0.221 | 0.1 | 0.016 | 0.115, 0.176 | 0.7 | 0.032 | 0.609, 0.733 |
| 2029 | 0.2 | 0.020 | 0.196, 0.274 | 0.2 | 0.020 | 0.154, 0.233 | 0.2 | 0.018 | 0.116, 0.186 | 0.7 | 0.036 | 0.619, 0.759 |
| 2030 | 0.2 | 0.022 | 0.196, 0.282 | 0.2 | 0.023 | 0.156, 0.245 | 0.2 | 0.020 | 0.117, 0.197 | 0.7 | 0.040 | 0.629, 0.785 |
| 2031 | 0.2 | 0.024 | 0.196, 0.291 | 0.2 | 0.026 | 0.158, 0.258 | 0.2 | 0.023 | 0.119, 0.207 | 0.7 | 0.044 | 0.639, 0.813 |
| 2032 | 0.2 | 0.026 | 0.195, 0.299 | 0.2 | 0.029 | 0.160, 0.272 | 0.2 | 0.025 | 0.120, 0.219 | 0.7 | 0.049 | 0.649, 0.841 |
| 2033 | 0.3 | 0.029 | 0.195, 0.308 | 0.2 | 0.032 | 0.162, 0.287 | 0.2 | 0.028 | 0.120, 0.231 | 0.8 | 0.054 | 0.659, 0.870 |
| 2034 | 0.3 | 0.031 | 0.194, 0.317 | 0.2 | 0.035 | 0.164, 0.302 | 0.2 | 0.031 | 0.121, 0.244 | 0.8 | 0.059 | 0.669, 0.900 |
| 2035 | 0.3 | 0.034 | 0.194, 0.326 | 0.2 | 0.039 | 0.166, 0.318 | 0.2 | 0.034 | 0.122, 0.257 | 0.8 | 0.064 | 0.679, 0.931 |
| 2036 | 0.3 | 0.036 | 0.193, 0.335 | 0.3 | 0.043 | 0.168, 0.334 | 0.2 | 0.038 | 0.123, 0.271 | 0.8 | 0.070 | 0.690, 0.964 |
| 2037 | 0.3 | 0.039 | 0.192, 0.345 | 0.3 | 0.047 | 0.169, 0.352 | 0.2 | 0.042 | 0.123, 0.286 | 0.8 | 0.076 | 0.700, 0.997 |
| 2038 | 0.3 | 0.042 | 0.191, 0.354 | 0.3 | 0.051 | 0.171, 0.370 | 0.2 | 0.045 | 0.123, 0.301 | 0.9 | 0.082 | 0.711, 1.031 |
| 2039 | 0.3 | 0.044 | 0.190, 0.364 | 0.3 | 0.055 | 0.172, 0.389 | 0.2 | 0.049 | 0.124, 0.317 | 0.9 | 0.088 | 0.721, 1.067 |
| 2040 | 0.3 | 0.047 | 0.189, 0.375 | 0.3 | 0.060 | 0.173, 0.409 | 0.2 | 0.054 | 0.124, 0.334 | 0.9 | 0.095 | 0.732, 1.103 |

All analyses were weighted using the provided discharge weights as recommended by HCUP. The variables “HOSP_NIS” and “NIS_Stratum” were used for clustering and stratification of the data, respectively.

Rates represent model-based fitted values for 2016-2020 and projected values for 2021-2040 derived from Poisson regression.

Standard Errors and Confidence Intervals are reported with sufficient decimal precision to avoid loss of information.

**Abbreviations:** None.

**Supplementary Table 6D.** **Prediction of** **cancer prevalence** in patients undergoing cardiovascular admissions until 2040, including Confidence Intervals and Standard Error.

| Years | Other Cancer  (IRR 1.038, 95% CI 1.023 – 1.053, P<0.001) | | |
| --- | --- | --- | --- |
|  | **Rate** | **Standard Error** | **[95% CI]** |
| 2016 | 0.2 | 0.004 | 0.199, 0.213 |
| 2017 | 0.2 | 0.003 | 0.209, 0.219 |
| 2018 | 0.2 | 0.002 | 0.218, 0.227 |
| 2019 | 0.2 | 0.003 | 0.225, 0.236 |
| 2020 | 0.2 | 0.004 | 0.231, 0.248 |
| 2021 | 0.2 | 0.006 | 0.237, 0.260 |
| 2022 | 0.3 | 0.008 | 0.243, 0.273 |
| 2023 | 0.3 | 0.010 | 0.248, 0.287 |
| 2024 | 0.3 | 0.012 | 0.254, 0.302 |
| 2025 | 0.3 | 0.015 | 0.260, 0.317 |
| 2026 | 0.3 | 0.017 | 0.265, 0.333 |
| 2027 | 0.3 | 0.020 | 0.271, 0.350 |
| 2028 | 0.3 | 0.023 | 0.277, 0.368 |
| 2029 | 0.3 | 0.026 | 0.283, 0.386 |
| 2030 | 0.3 | 0.030 | 0.289, 0.406 |
| 2031 | 0.4 | 0.034 | 0.295, 0.426 |
| 2032 | 0.4 | 0.038 | 0.300, 0.448 |
| 2033 | 0.4 | 0.042 | 0.306, 0.470 |
| 2034 | 0.4 | 0.046 | 0.312, 0.493 |
| 2035 | 0.4 | 0.051 | 0.318, 0.518 |
| 2036 | 0.4 | 0.056 | 0.324, 0.544 |
| 2037 | 0.5 | 0.061 | 0.330, 0.570 |
| 2038 | 0.5 | 0.067 | 0.336, 0.599 |
| 2039 | 0.5 | 0.073 | 0.342, 0.628 |
| 2040 | 0.5 | 0.079 | 0.348, 0.659 |

All analyses were weighted using the provided discharge weights as recommended by HCUP. The variables “HOSP_NIS” and “NIS_Stratum” were used for clustering and stratification of the data, respectively.

Rates represent model-based fitted values for 2016-2020 and projected values for 2021-2040 derived from Poisson regression.

Standard Errors and Confidence Intervals are reported with sufficient decimal precision to avoid loss of information.

**Abbreviations:** None.

**Supplementary Figure 1A-F.** Prediction of prevalence specific cancers in patients undergoing cardiovascular admissions until 2040 – (A) Breast, (B) colorectal, (C) renal, (D) liver, (E) haematological, (F) lung cancer.

**
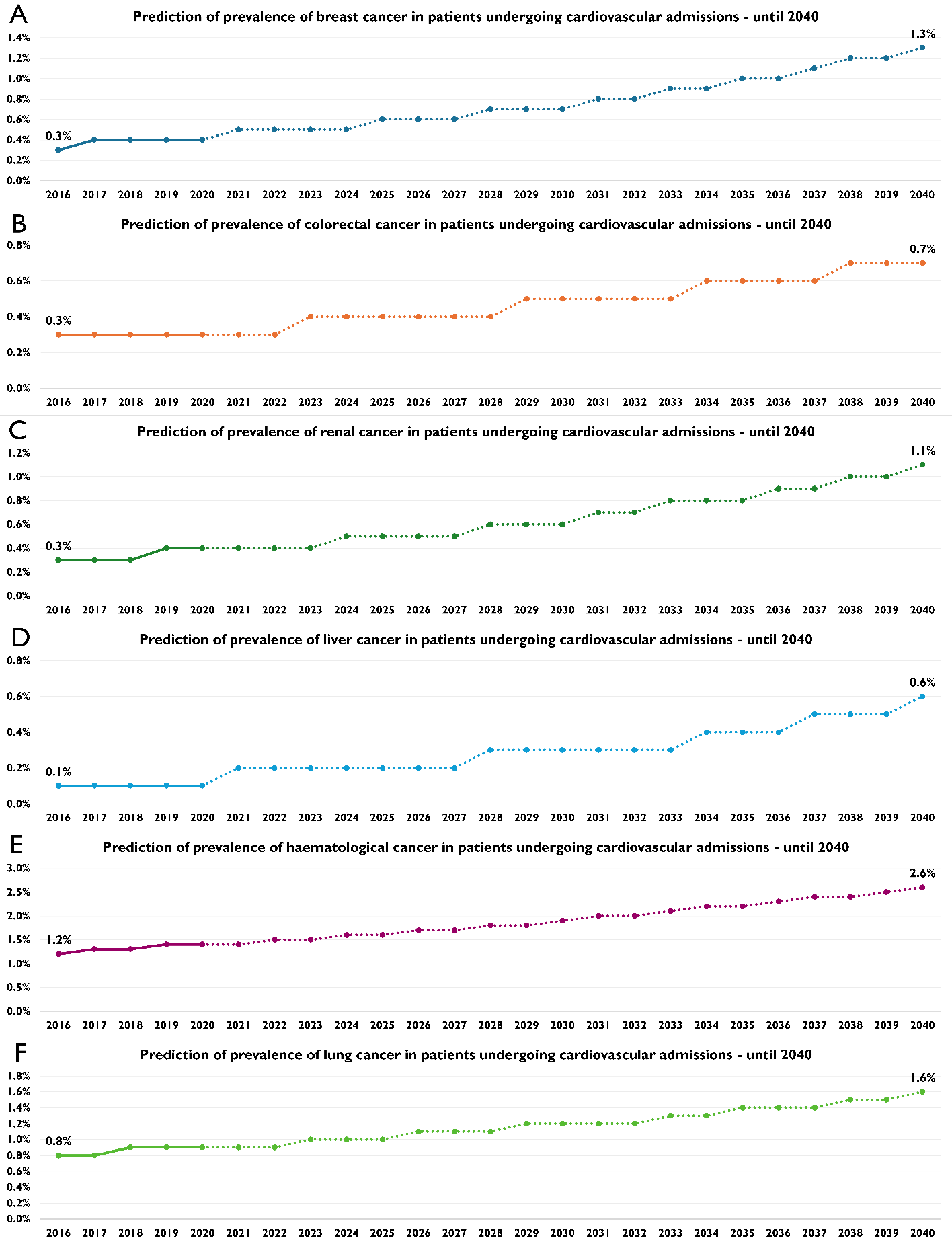
**

The solid line represents baseline data, while the dotted line represents projected data.

**Abbreviations:** None

**Supplementary Figure 2A-G.** Prediction of prevalence specific cancers in patients undergoing cardiovascular admissions until 2040 – (A) Prostate and male genital, (B) pancreatic, (C) female genital, (D) skin, (E) gastroesophageal, (F) secondary unspecified and (G) other cancers.

**
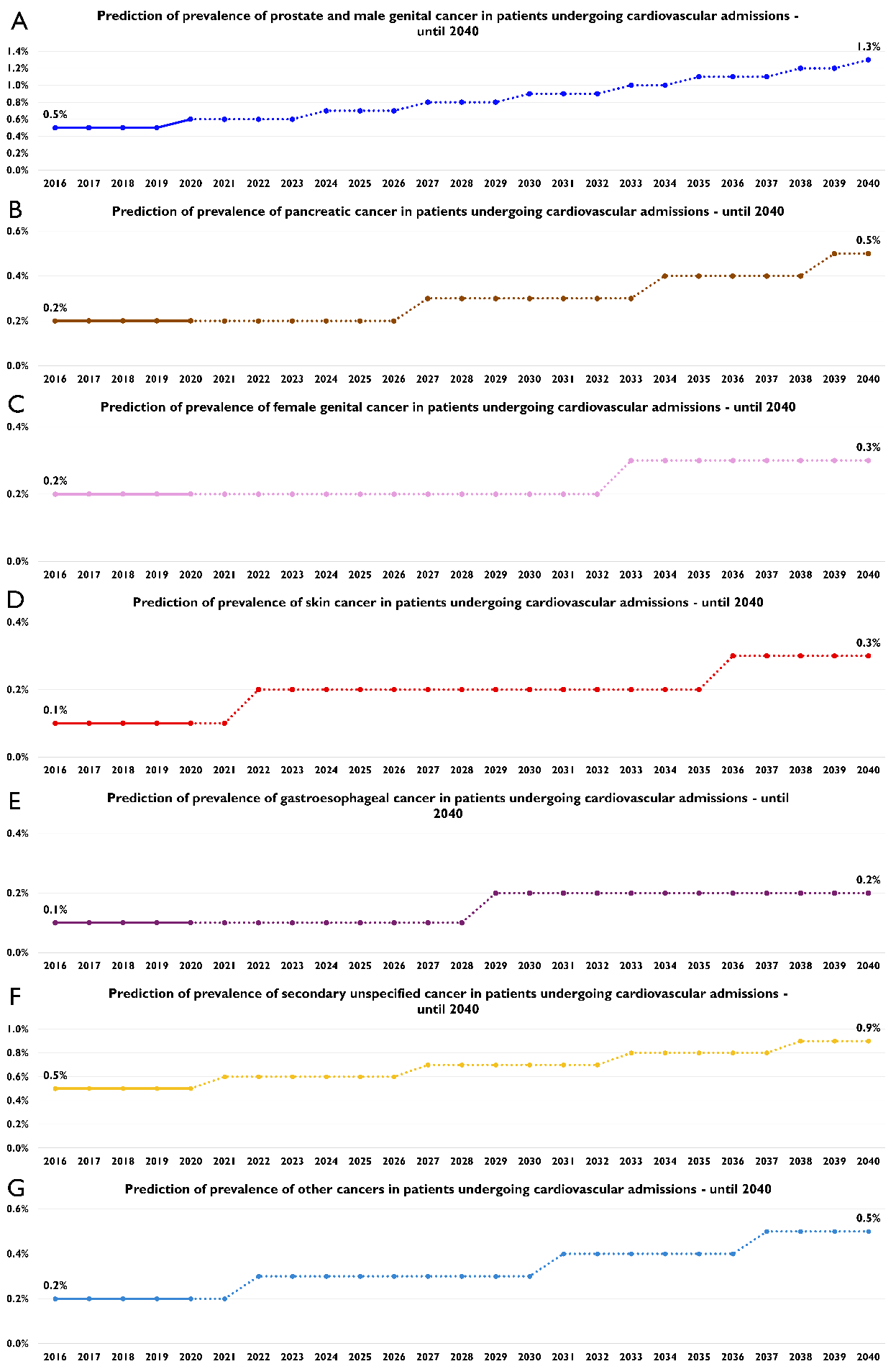
**

The solid line represents baseline data, while the dotted line represents projected data.

**Abbreviations:** None.

**Supplementary Figure 3.** Prevalence of breast cancer based on specific cardiovascular admission causes across study period.


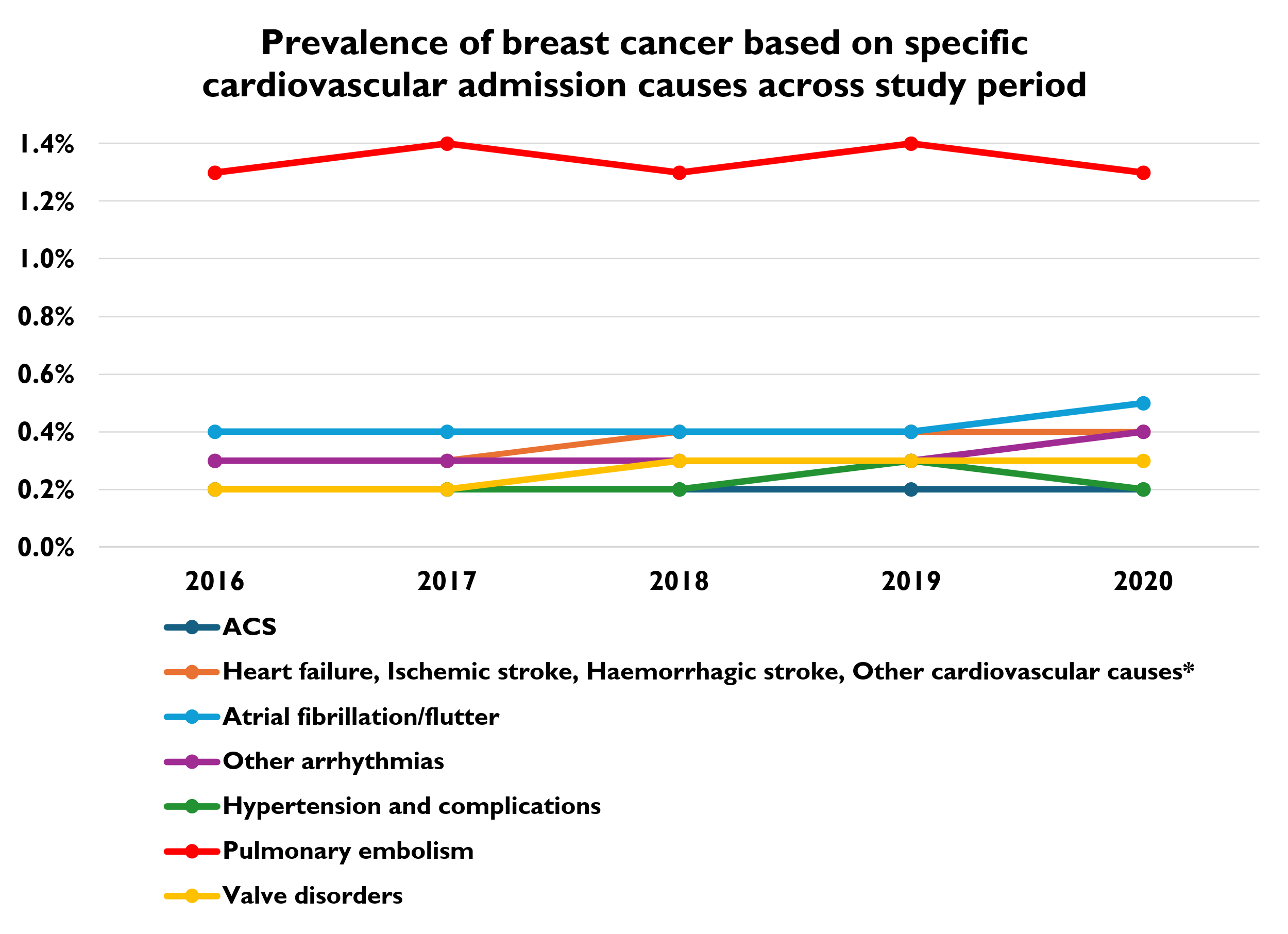


*A single line is displayed where prevalence was identical across admission categories during the study period.

**Abbreviations:** None.

**Supplementary Figure 4.** Prevalence of colorectal cancer based on specific cardiovascular admission causes across study period.


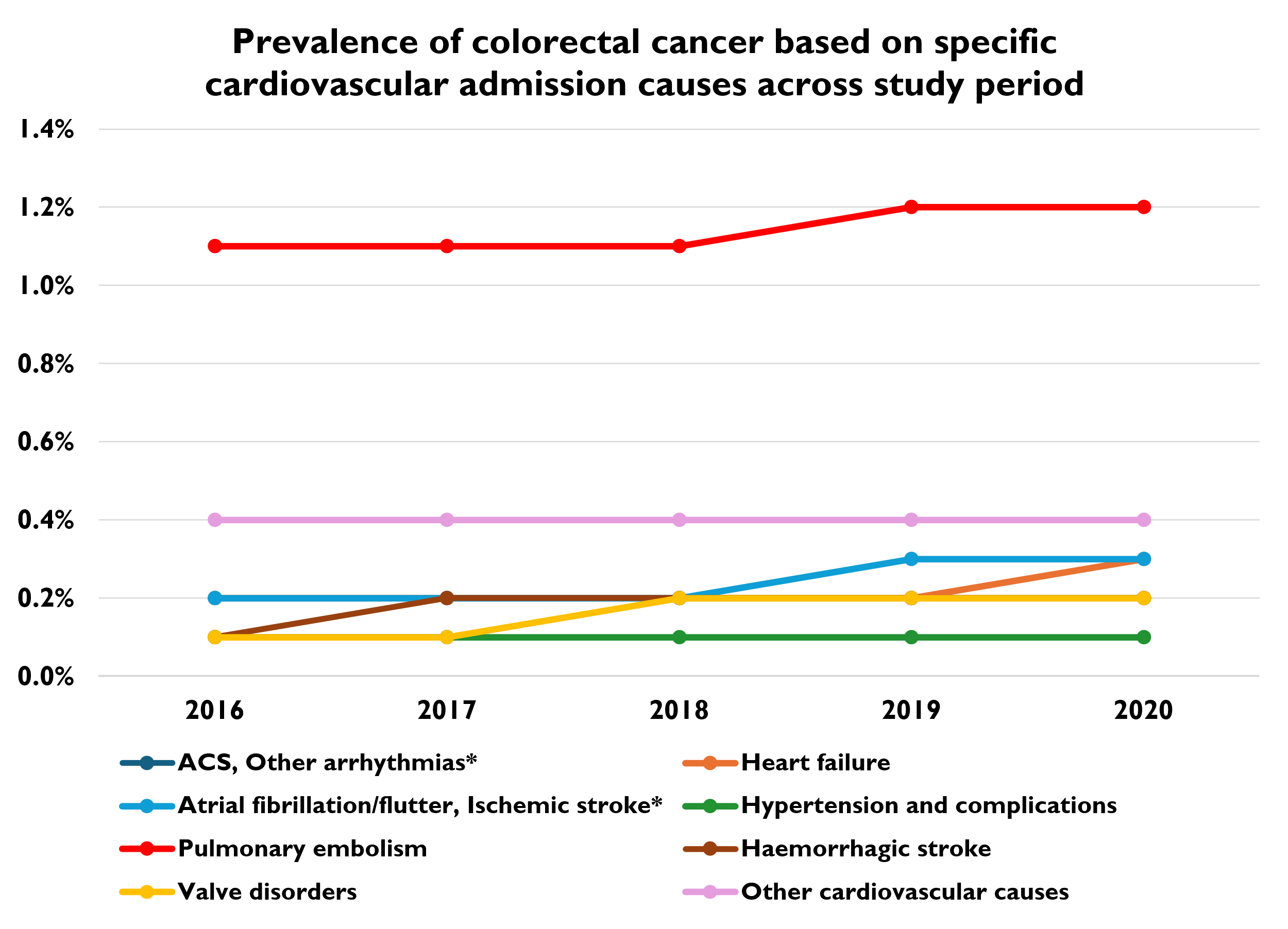


*A single line is displayed where prevalence was identical across admission categories during the study period.

**Abbreviations:** None.

**Supplementary Figure 5.** Prevalence of renal cancer based on specific cardiovascular admission causes across study period.


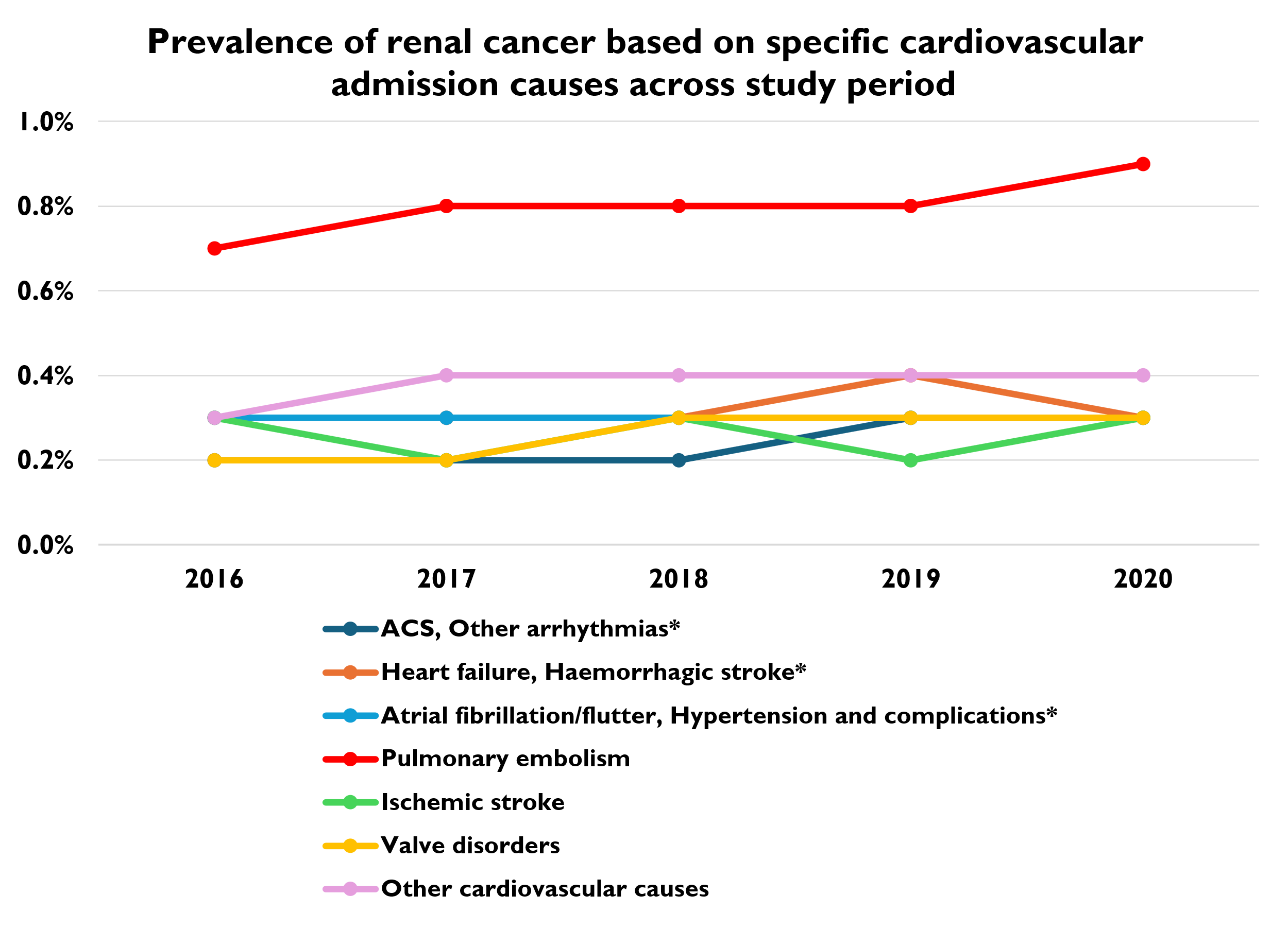


*A single line is displayed where prevalence was identical across admission categories during the study period.

**Abbreviations:** None.

**Supplementary Figure 6.** Prevalence of liver cancer based on specific cardiovascular admission causes across study period.


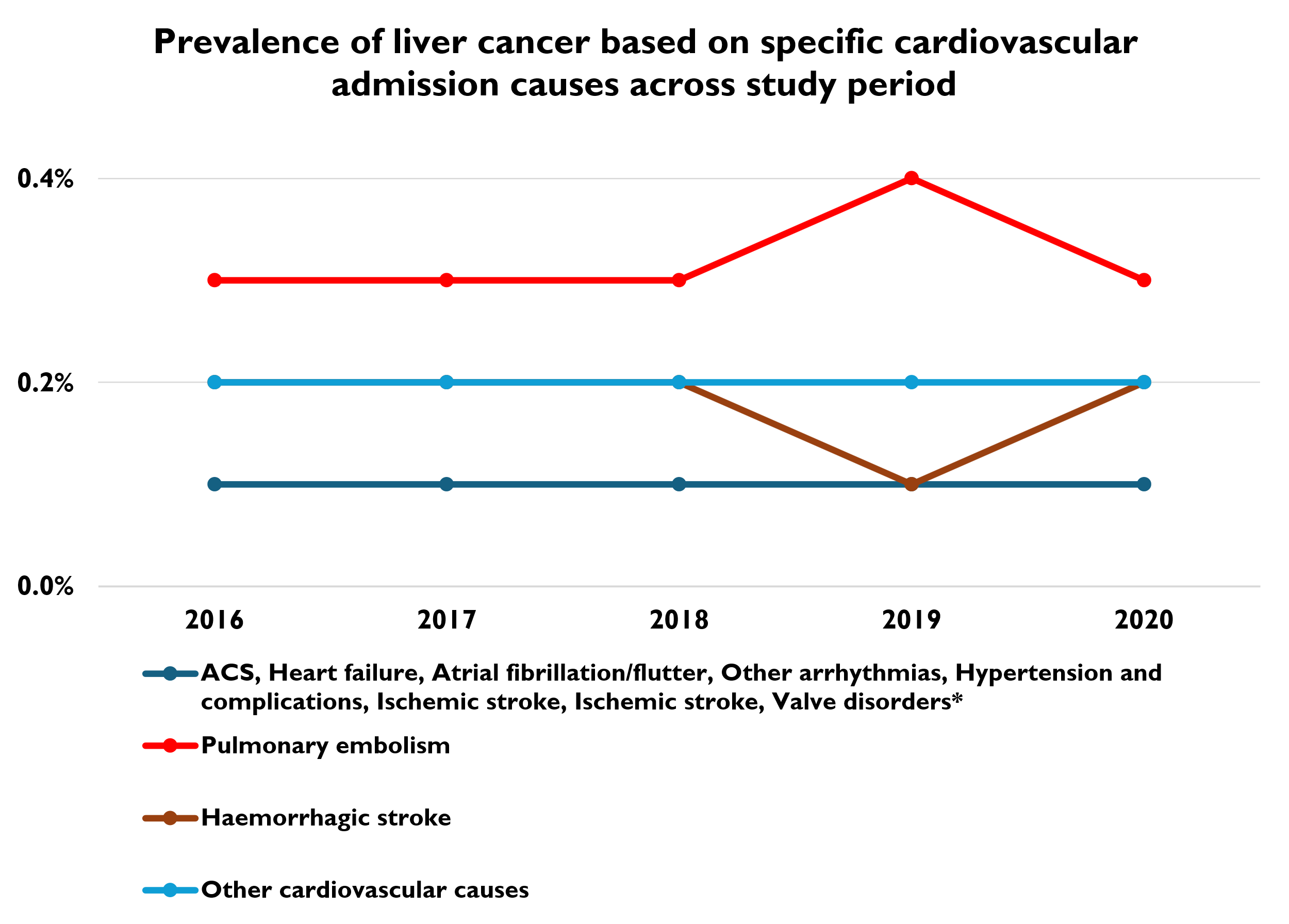


*A single line is displayed where prevalence was identical across admission categories during the study period.

**Abbreviations:** None.

**Supplementary Figure 7.** Prevalence of haematological cancer based on specific cardiovascular admission causes across study period.


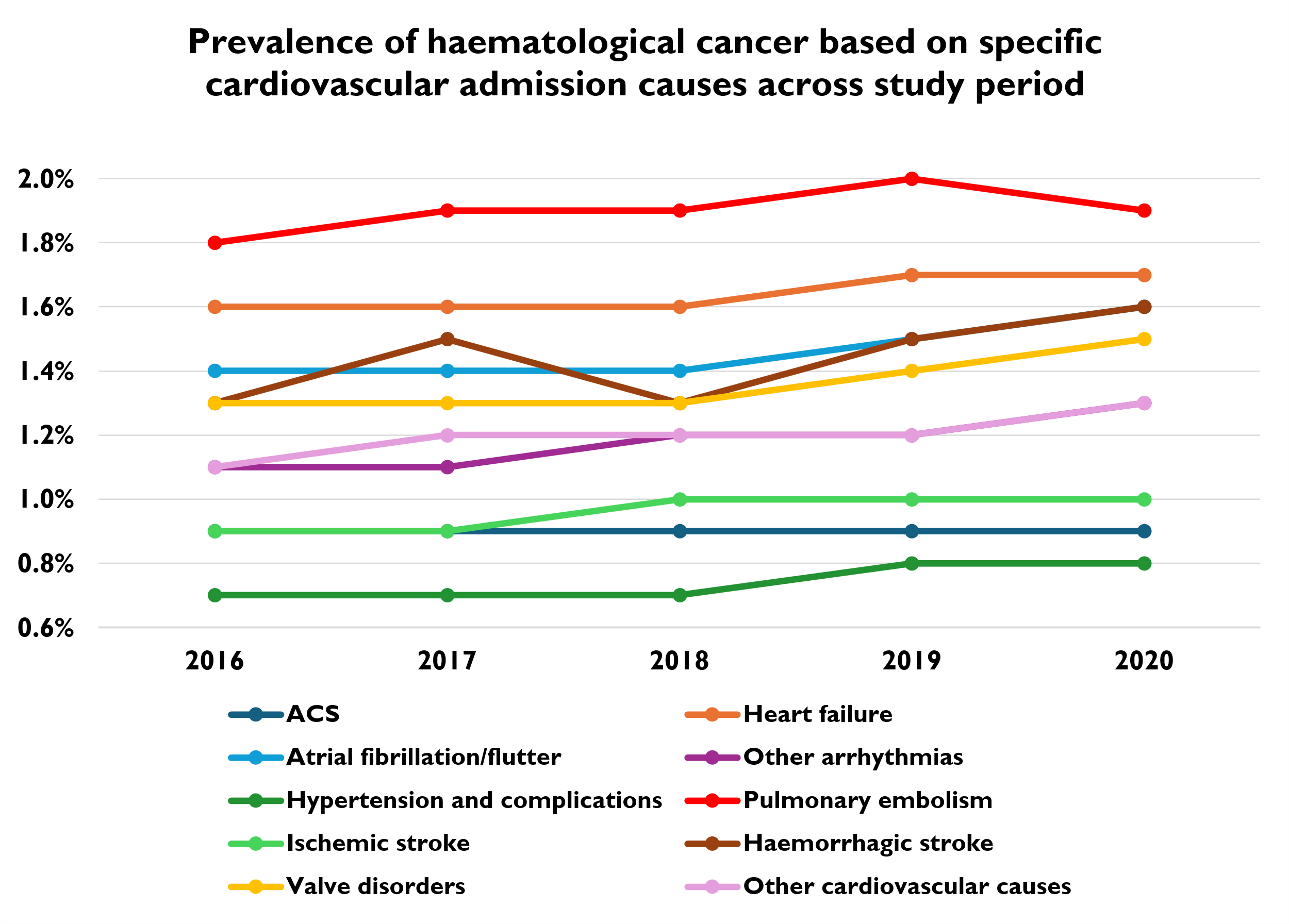


**Abbreviations:** None.

**Supplementary Figure 8.** Prevalence of lung cancer based on specific cardiovascular admission causes across study period.


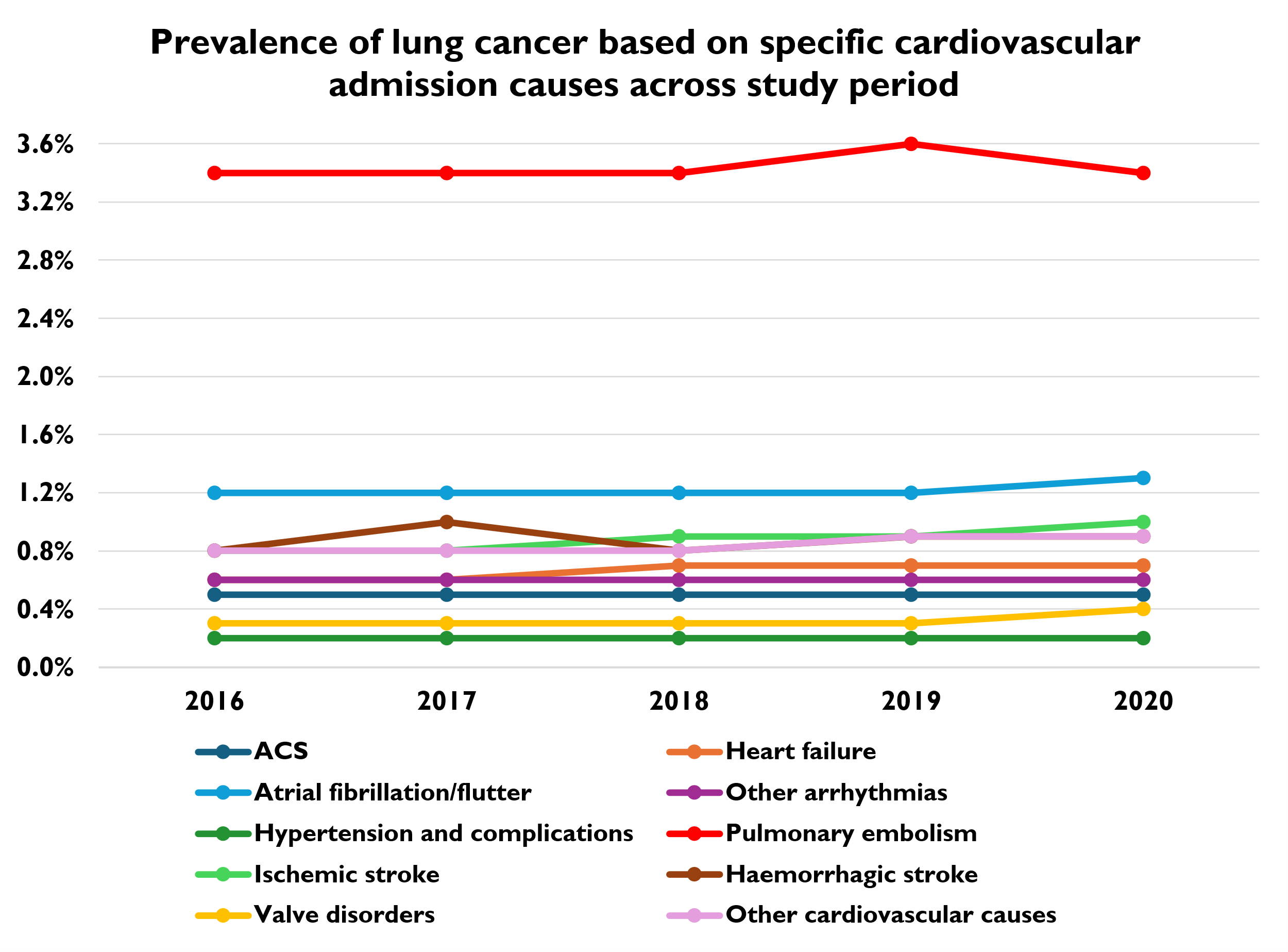


**Abbreviations:** None.

**Supplementary Figure 9.** Prevalence of prostate and male genital cancer based on specific cardiovascular admission causes across study period.


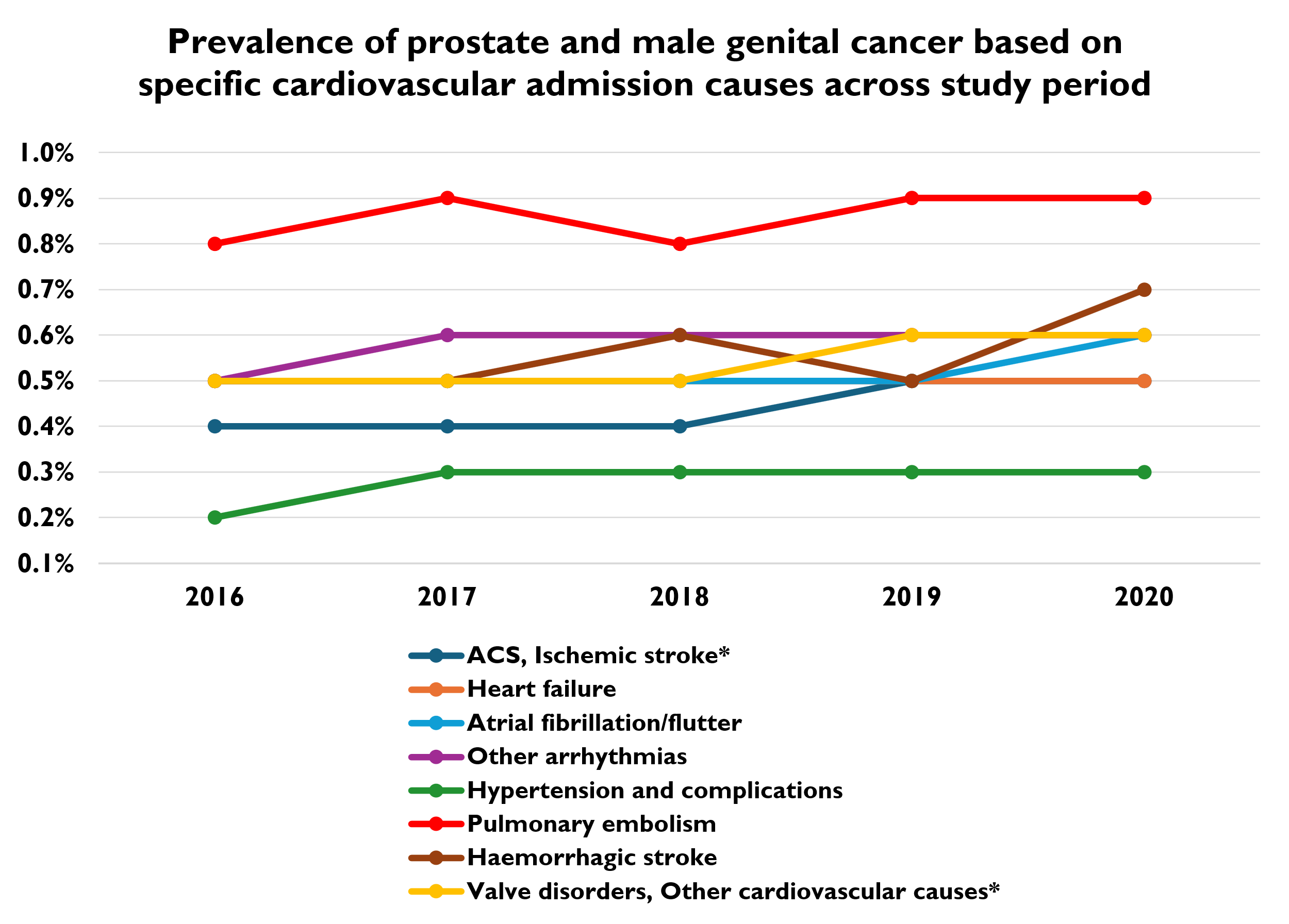


*A single line is displayed where prevalence was identical across admission categories during the study period.

**Abbreviations:** None.

**Supplementary Figure 10.** Prevalence of pancreatic cancer based on specific cardiovascular admission causes across study period.


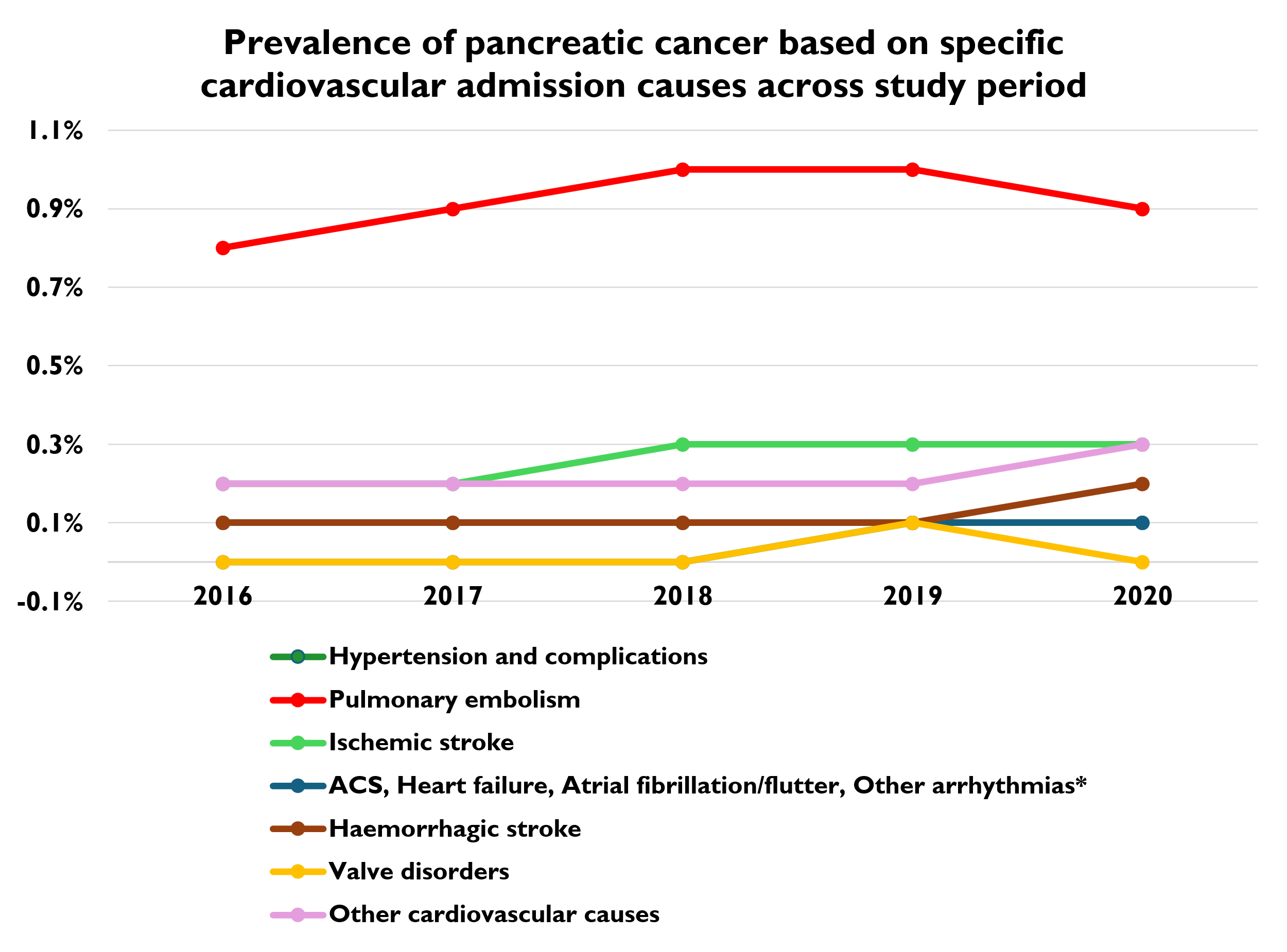


*A single line is displayed where prevalence was identical across admission categories during the study period.

**Abbreviations:** None.

**Supplementary Figure 11.** Prevalence of female genital cancer based on specific cardiovascular admission causes across study period.


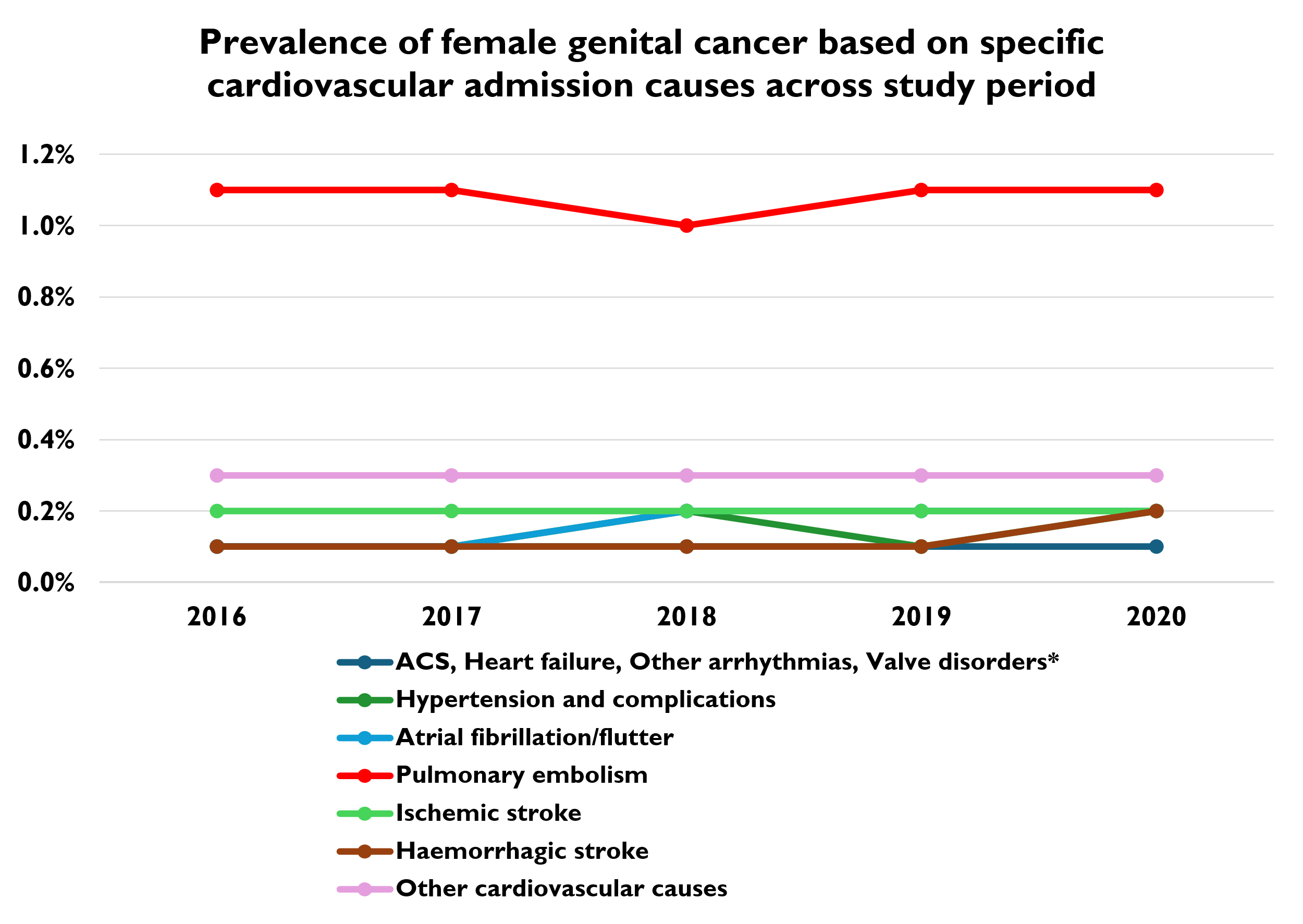


*A single line is displayed where prevalence was identical across admission categories during the study period.

**Abbreviations:** None.

**Supplementary Figure 12.** Prevalence of skin cancer based on specific cardiovascular admission causes across study period.


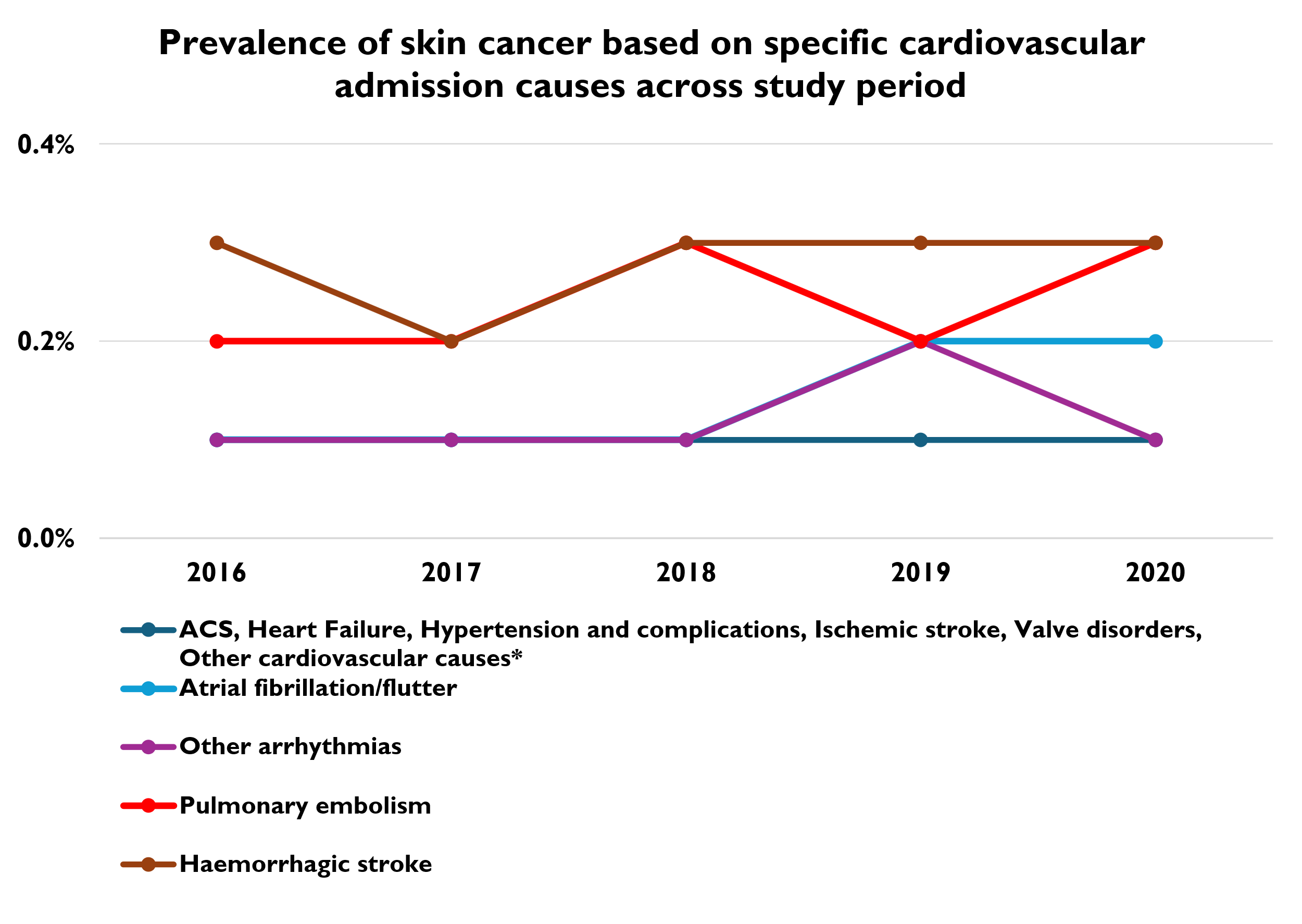


*A single line is displayed where prevalence was identical across admission categories during the study period.

**Abbreviations:** None.

**Supplementary Figure 13.** Prevalence of gastroesophageal cancer based on specific cardiovascular admission causes across study period.


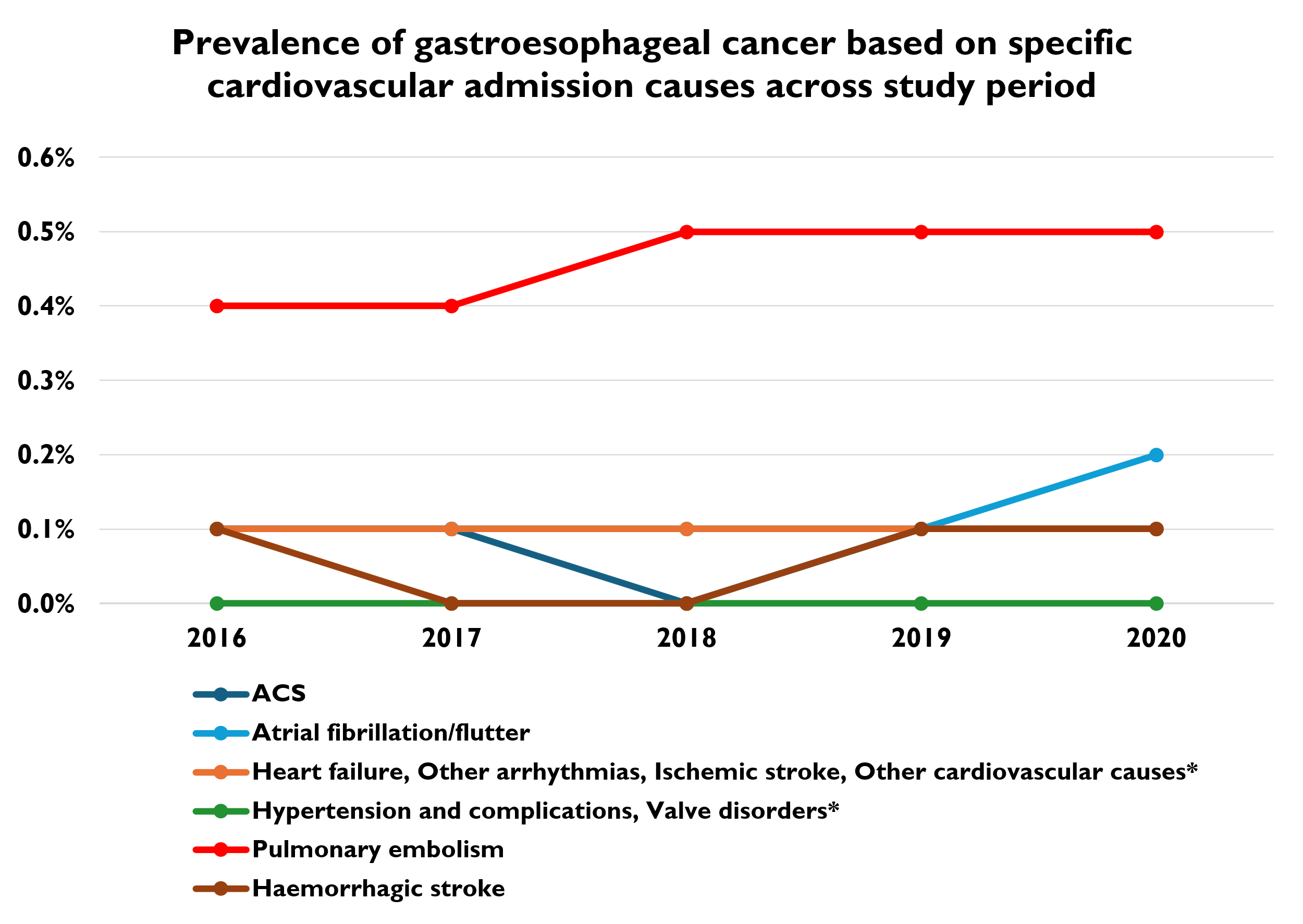


*A single line is displayed where prevalence was identical across admission categories during the study period.

**Abbreviations:** None.

**Supplementary Figure 14.** Prevalence of secondary unspecified cancer based on specific cardiovascular admission causes across study period.


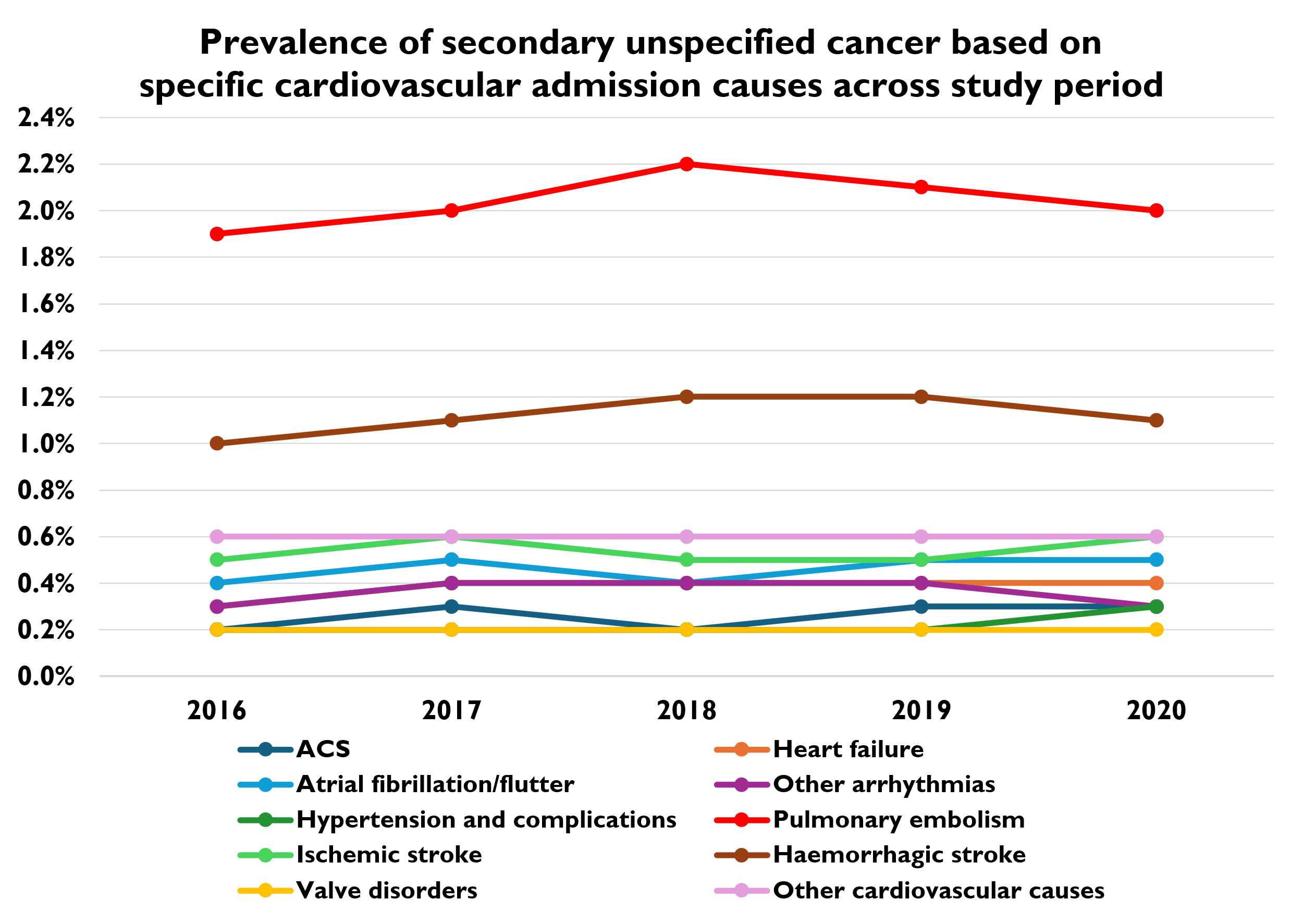


*A single line is displayed where prevalence was identical across admission categories during the study period.

**Abbreviations:** None.

**Supplementary Figure 15.** Prevalence of other cancers based on specific cardiovascular admission causes across study period.


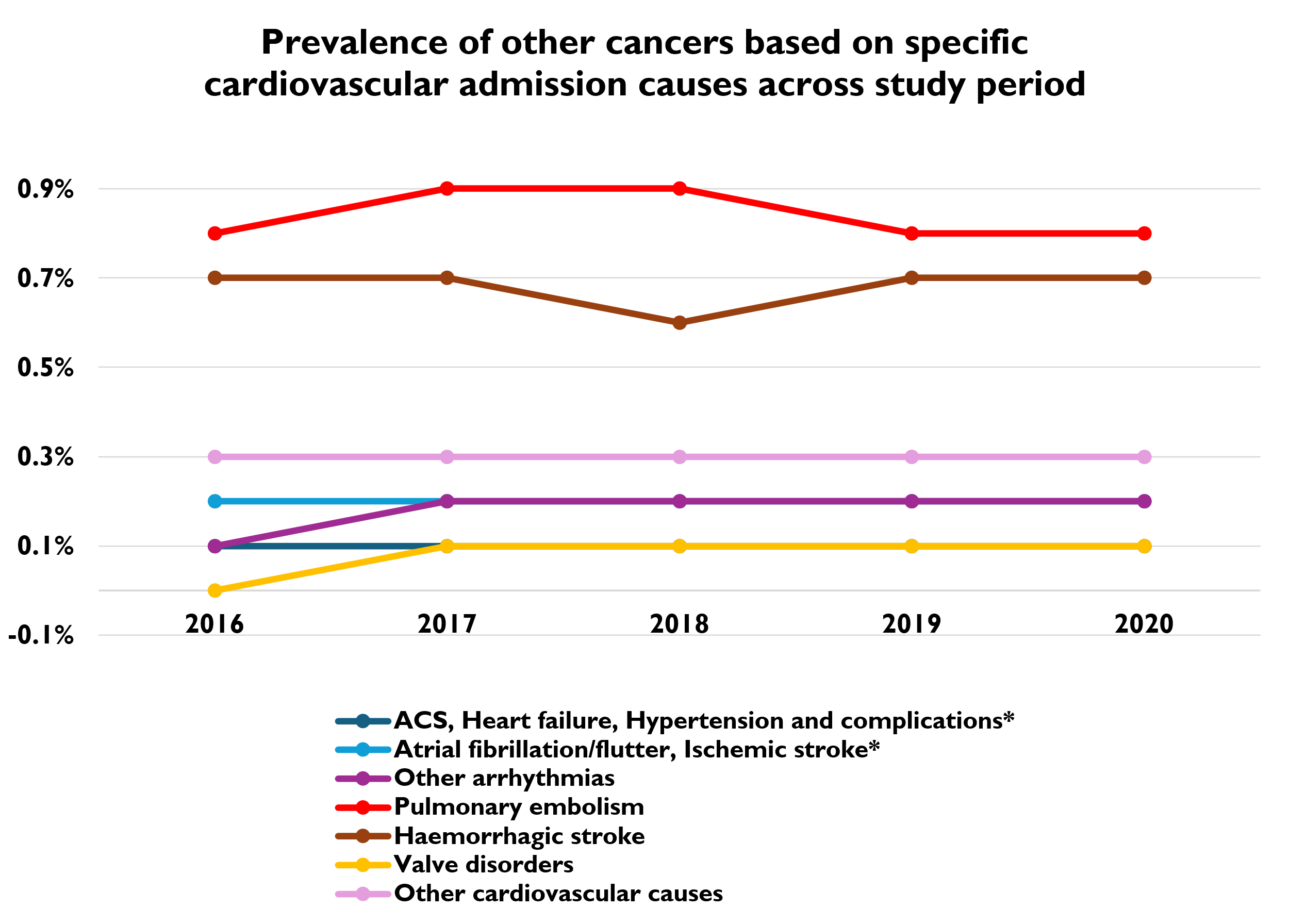


*A single line is displayed where prevalence was identical across admission categories during the study period.

**Abbreviations:** None.
